# The tale of TILs in breast cancer: A report from The International Immuno-Oncology Biomarker Working Group

**DOI:** 10.1038/s41523-021-00346-1

**Published:** 2021-12-01

**Authors:** Khalid El Bairi, Harry R. Haynes, Elizabeth Blackley, Susan Fineberg, Jeffrey Shear, Sophia Turner, Juliana Ribeiro de Freitas, Daniel Sur, Luis Claudio Amendola, Masoumeh Gharib, Amine Kallala, Indu Arun, Farid Azmoudeh-Ardalan, Luciana Fujimoto, Luz F. Sua, Shi-Wei Liu, Huang-Chun Lien, Pawan Kirtani, Marcelo Balancin, Hicham El Attar, Prerna Guleria, Wenxian Yang, Emad Shash, I-Chun Chen, Veronica Bautista, Jose Fernando Do Prado Moura, Bernardo L. Rapoport, Carlos Castaneda, Eunice Spengler, Gabriela Acosta-Haab, Isabel Frahm, Joselyn Sanchez, Miluska Castillo, Najat Bouchmaa, Reena R. Md Zin, Ruohong Shui, Timothy Onyuma, Wentao Yang, Zaheed Husain, Karen Willard-Gallo, An Coosemans, Edith A. Perez, Elena Provenzano, Paula Gonzalez Ericsson, Eduardo Richardet, Ravi Mehrotra, Sandra Sarancone, Anna Ehinger, David L. Rimm, John M. S. Bartlett, Giuseppe Viale, Carsten Denkert, Akira I. Hida, Christos Sotiriou, Sibylle Loibl, Stephen M. Hewitt, Sunil Badve, William Fraser Symmans, Rim S. Kim, Giancarlo Pruneri, Shom Goel, Prudence A. Francis, Gloria Inurrigarro, Rin Yamaguchi, Hernan Garcia-Rivello, Hugo Horlings, Said Afqir, Roberto Salgado, Sylvia Adams, Marleen Kok, Maria Vittoria Dieci, Stefan Michiels, Sandra Demaria, Sherene Loi, The International Immuno-Oncology Biomarker Working Group, Khalid El Bairi, Harry R. Haynes, Elizabeth Blackley, Susan Fineberg, Jeffrey Shear, Sophia Turner, Juliana Ribeiro de Freitas, Daniel Sur, Luis Claudio Amendola, Masoumeh Gharib, Amine Kallala, Indu Arun, Farid Azmoudeh-Ardalan, Luciana Fujimoto, Luz F. Sua, Shi-Wei Liu, Huang-Chun Lien, Pawan Kirtani, Marcelo Balancin, Hicham El Attar, Prerna Guleria, Wenxian Yang, Emad Shash, I-Chun Chen, Veronica Bautista, Jose Fernando Do Prado Moura, Bernardo L. Rapoport, Carlos Castaneda, Eunice Spengler, Gabriela Acosta-Haab, Isabel Frahm, Joselyn Sanchez, Miluska Castillo, Najat Bouchmaa, Reena R. Md Zin, Ruohong Shui, Timothy Onyuma, Wentao Yang, Zaheed Husain, Karen Willard-Gallo, An Coosemans, Edith A. Perez, Elena Provenzano, Paula Gonzalez Ericsson, Eduardo Richardet, Ravi Mehrotra, Sandra Sarancone, Anna Ehinger, David L. Rimm, John M. S. Bartlett, Giuseppe Viale, Carsten Denkert, Akira I. Hida, Christos Sotiriou, Sibylle Loibl, Stephen M. Hewitt, Sunil Badve, William Fraser Symmans, Rim S. Kim, Giancarlo Pruneri, Shom Goel, Prudence A. Francis, Gloria Inurrigarro, Rin Yamaguchi, Hernan Garcia-Rivello, Hugo Horlings, Said Afqir, Roberto Salgado, Sylvia Adams, Marleen Kok, Maria Vittoria Dieci, Stefan Michiels, Sandra Demaria, Sherene Loi, Vera Schelfhout, Elham Arbzadeh, Anastasiya Bondanar, Silvio Antonio Galeano Reyes, Jose Ramirez Ruz, Jun Kang, Lu Xiang, Martina Zimovjanova, Pilar Togores, Tulin Ozturk, Asawari Patil, Marcus Corpa, Ann Whitehouse, Benjamin Tan, Alfredo de Paula, Claudia Rossetti, Corinna Lang-Schwarz, Sarah Mahon, Cinzia Giacometti, Barbro Linderholm, Frederik Deman, Giacomo Montagna, Gyungyub Gong, Marta Pavcovich, Yeesoo Chaer, Isabel Alvarado Cabrero, Mayana Lopes de Brito, Nevena Ilieva, Annamaria Fulop, Maiara Souza, Domenico Bilancia, Michael Idowu, Ritika Johri, Joanna Szpor, Lira Bachani, Fernando Schmitt, Mag Giannotti, Yutaka Kurebayashi, Bruno Elias Anota Ramirez, Eduardo Salido, Laura Bortesi, Sara Bonetto, Kevin Elomina, Patricia Lopez, Vijay Sharma, Amalika Edirisinghe, Dhanvi Mathur, Ayushi Sahay, Makhlouf Ait Mouloud, Chau Huynh Giang, Edwin Mukolwe, Edgar Kiruka, Nancy Samberg, Norie Abe, Mark Brown, Ewan Millar, Xiaoxian (Bill) Li, Zheng Yuan, Asokan Pasupathy, Raffaele Miele, Ronald Luff, Monica Modesto Araujo e Porfirio, Ogugua Ajemba, Rashida Soni, Enrico Orvieto, Michael DiMaio, Jeremy Thomas, Reena Merard, Manish Mani Subramaniam, Thiago Apolinario, Ovidiu Preda, Ricardo Preda, Alexander Makanga, Marcelo Souto Maior, Lingyu Li, Mahasti Saghatchian, Tricia Saurine, Emiel Janssen, John Cochran, Nikitina Vlada, Rocco Cappellesso, Katherine Elfer, Morven Hollick, Sangeeta Desai, Gizem Oner, Arthur Schreurs, Steve Liu, Rashindrie Perera, Paola Mercurio, Felip Garcia, Kareem Hosny, Hirofumi Matsumoto, Carolien van Deurzen, Giampaolo Bianchini, Ipek Coban, Arif Jahangir, Arman Rahman, Daniel Stover, Paulo Luz, Anne Martel, Yannick Waumans, Albrecht Stenzinger, Javier Cortes, Polina Dimitrova, Inne Nauwelaers, Montse Velasco, Fang Fan, Guray Akturk, Michael Firer, Ioannis Roxanis, Mary Schneck, Hannah Wen, Vincent Cockenpot, Aleksei Konstantinov, Ana Calatrava, M. N. Vidya, Hyun Joo Choi, Paul Jank, Aini Hyyti ÇÏinen, Dhanusha Sabanathan, Giuseppe Floris, Doris Hoeflmayer, Tetsuo Hamada, Nele Laudus, Anita Grigoriadis, Ilaria Porcellato, Balazs Acs, Federica Miglietta, Jeannette Parrodi, David Clunie, Benjamin Calhoun, Fang-I Lu, Alex Lefevre, Sami Tabbarah, William Tran, Isaac Garcia-murillas, Petar Jelinic, Carolien Boeckx, Sandra Souza, MarÇða Cebollero, Eudald Felip, Jose Luis Solorzano Rendon, Ehab El Gabry, Joel Saltz, Emilio Bria, Giovanna Garufi, Johan Hartman, Manu Sebastian, Helena Olofsson, Loes Kooreman, Joël Cucherousset, Marie-Christine Mathieu, Carmen Ballesteros-Merino, Popi Siziopikou, Jacinta Fong, Molly Klein, Ignasi Roig I. Qulis, Jelle Wesseling, Enrique Bellolio, Juan Carlos Araya, Stephen Naber, Maggie Cheang, Isabella Castellano, Ales Ales, Anne-Vibeke Laenkholm, Janina Kulka, Cecily Quinn, Anna Sapino, Isabel Amendoeira, Caterina Marchio, Jeremy Braybrooke, Anne Vincent-Salomon, Konstanty (Popi) Korski, Michail Sofopoulos, Elisabeth Ida Specht Stovgaard, Simonetta Bianchi, Zsuzsanna Bago-Horvath, Clare Yu, Peter Regitnig, Sean Hall, Zuzana Kos, Sneha Sant, Jean-Christophe Tille, Brandon Gallas, Daniel Bethmann, Peter Savas, Larissa Mendes, Teresa Soler, Maartje van Seijen, Tina Gruosso, Angela Quintana, Jennifer Giltnane, Gert Van den Eynden, Eleonora Duregon, Rafa de Cabo, Phil Coates Recamo, Louis Gaboury, Johannes Zimmerman, Claudia Stanciu Pop, Alejandra Wernicke, David Williams, Anthony Gill, Benjamin Solomon, Bibhusal Thapa, Gelareh Farshid, Leslie Gilham, Michael Christie, Sandra O’Toole, Shona Hendry, Stephen B. Fox, Stephen J. Luen, Sunil R. Lakhani, Talia Fuchs, Tom John, Iva Brcic, Johannes Hainfellner, Lax Sigurd, Matthias Preusser, Philip Poortmans, Alex Decaluwe, Caroline Carey, Cecile Colpaert, Denis Larsimont, Dieter Peeters, Glenn Broeckx, Koen van de Vijver, Laurence Buisseret, Luc Dirix, Marjan Hertoghs, Martine Piccart, Michail Ignatiadis, Mieke Van Bockstal, Nicolas Sirtaine, Peter Vermeulen, Roland de Wind, Sabine Declercq, Thomas Gevaert, Benjamin Haibe-Kans, Brad H. Nelson, Peter H. Watson, Sam Leung, Torsten Nielsen, Leming Shi, Eva Balslev, Jeppe Thagaard, Alhadi Almangush, Antti Makitie, Heikki Joensuu, Johan Lundin, Damien Drubay, Elvire Roblin, Fabrice Andre, Frederique Penault-Llorca, Jerome Lemonnier, Julien Adam, Magali Lacroix-Triki, Nils Ternes, Nina Radosevic-Robin, Frederick Klaushen, Karsten Weber, Nadia Harbeck, Oleg Gluz, Stephan Wienert, Gabor Cserni, Andrea Vingiani, Carmen Criscitiello, Cinzia Solinas, Giuseppe Curigliano, Eiichi Konishi, Eiji Suzuki, Katsuhiro Yoshikawa, Kosuke Kawaguchi, Masahiro Takada, Masakazu Toi, Mitsuaki Ishida, Nobuhiro Shibata, Shigehira Saji, Takahiro Kogawa, Takashi Sakatani, Takeru Okamoto, Takuya Moriya, Tatsuki Kataoka, Tatsunori Shimoi, Tomohagu Sugie, Tomoharu Sugie, Toru Mukohara, Yazaki Shu, Yuichiro Kikawa, Yuji Kozuka, Shahin Sayed, Reena Rahayu, Reena Ramsaroop, Elżbieta Senkus-Konefka, Ewa Chmielik, Fatima Cardoso, Joana Ribeiro, Jack Chan, Rebecca Dent, Miguel Martin, Carlos Hagen, Angel Guerrero, Federico Rojo, Laura Comerma, Paolo Nuciforo, Victor Vivo Serrano, Vincente Peg Cámaea, Tessa Steenbruggen, Francesco Ciompi, Iris Nederlof, Jan Hudecek, Jeroen van der Laak, Jose van den Berg, Leonie Voorwerk, Mark van de Vijver, Michiel de Maaker, Sabine Linn, Hayley McKenzie, Navita Somaiah, Andrew Tutt, Charles Swanton, Crispin Hiley, David A. Moore, Jacqueline A. Hall, John Le Quesne, Khalid Abdul Jabbar, Maise al Bakir, Robert Hills, Sheeba Irshad, Yinyin Yuan, Zaibo Li, Minetta Liu, Jonathan Klein, Oluwole Fadare, Alastair Thompson, Alexander J. Lazar, Allen Gown, Amy Lo, Ana C. Garrido Castro, Anant Madabhushi, Andre Moreira, Andrea Richardson, Andrew H. Beck, Andrew M. Bellizzi, Antonio Wolff, Aparna Harbhajanka, Ashish Sharma, Ashley Cimino-Mathews, Ashok Srinivasan, Baljit Singh, Chakra S. Chennubhotla, Cynthia Chauhan, Deborah A. Dillon, Dimitrios Zardavas, Douglas B. Johnson, Aubrey E. Thompson, Edi Brogi, Emily Reisenbichler, Erich Huang, Fred R. Hirsch, Heather McArthur, James Ziai, Jane Brock, Jennifer Kerner, Jiping Zha, Jochen K. Lennerz, Jodi M. Carter, Jorge Reis-Filho, Joseph Sparano, Justin M. Balko, Katherine Pogue-Geile, Keith E. Steele, Kim R. M. Blenman, Kimberly H. Allison, Lajos Pusztai, Lee Cooper, Valeria M. Estrada, Margaret Flowers, Mark Robson, Marlon C. Rebelatto, Matthew G. Hanna, Matthew P. Goetz, Mehrnoush Khojasteh, Melinda E. Sanders, Meredith M. Regan, Michael Misialek, Mohamed Amgad, Nadine Tung, Rajendra Singh, Richard Huang, Robert H. Pierce, Roberto Leon-Ferre, Sandra Swain, Scott Ely, Seong-Rim Kim, Shahinaz Bedri, Soonmyung Paik, Stuart Schnitt, Timothy d’Alfons, Uday Kurkure, Veerle Bossuyt, Weida Tong, Yihong Wang, Carlos Henrique Dos Anjos, Fabien Gaire, Paul J. Van Diest

**Affiliations:** 1 Department of Medical Oncology, Mohammed VI University Hospital, Faculty of Medicine and Pharmacy, Mohammed Ist University, Oujda, Morocco; 2 Department of Cellular Pathology, Great Western Hospital, Swindon, UK; 3 Translational Health Sciences, University of Bristol, Bristol, UK; 4 Division of Research, Peter MacCallum Cancer Centre, Melbourne, Australia; 5 Department of Pathology, Montefiore Medical Center and the Albert Einstein College of Medicine, Bronx, NY USA; 6 Chief Information Officer, WISS & Company, LLP and President J. Shear Consulting, LLC–Ardsley, Ardsley, NY USA; 7 ICPV, Independent Cancer Patient Voice, London, UK; 8 Department of Pathology and Legal Medicine, Medical School of the Federal University of Bahia, Salvador, Brazil; 9 Department of Medical Oncology, University of Medicine “I. Hatieganu”, Cluj Napoca, Romania; 10 Brazilian Society of Oncology, Salvador, Brazil; 11 Faculty of Medicine, Mashhad University of Medical Sciences, Mashhad, Iran; 12 Hopital Charles Nicolle, Tunis, Tunisia; 13 Department of Histopathology, Tata Medical Center, Kolkata, India; 14 Department of Pathology, Imam Khomeini Hospital Complex, Tehran University of Medical Sciences, Tehran, Iran; 15 Pathology and Legal Medicine, Amazon Federal University, Belém, Brazil; 16 Department of Pathology and Laboratory Medicine, Fundacion Valle del Lili, and Faculty of Health Sciences, Universidad ICESI, Cali, Colombia; 17 Sichuan Cancer Hospital, Chengdu, China; 18 Department of Pathology, National Taiwan University Hospital, Taipei, Taiwan; 19 Department of Histopathology, Manipal Hospitals Dwarka, New Delhi, India; 20 Department of Pathology, Faculty of Medicine, University of São Paulo, São Paulo, Brazil; 21 Apc labs - Annasr Pathology Center, El Jadida, Morocco; 22 Army Hospital Research and Referral, Delhi Cantt, New Delhi, India; 23 Aginome Scientific Pte Ltd, Xiamen, China; 24 Breast Cancer Comprehensive Center, National Cancer Institute, Cairo University, Cairo, Egypt; 25 Department of Oncology, National Taiwan University Cancer Center, Taipei, Taiwan; 26 Department of Oncology, National Taiwan University Hospital, Taipei, Taiwan; 27 Department of Pathology, Breast Cancer Center FUCAM, Mexico City, Mexico; 28 Centro de Pesquisas Clinicas do IMIP, Recife, Brazil; 29 The Medical Oncology Centre of Rosebank, Johannesburg, South Africa; 30 Department of Immunology, Faculty of Health Sciences, University of Pretoria, corner Doctor Savage Road and Bophelo Road, Pretoria, 0002 South Africa; 31 Department of Medical Oncology, Instituto Nacional de Enfermedades Neoplásicas, Lima, 15038 Peru; 32 Faculty of Health Sciences, Universidad Cientifica del Sur, Lima, Peru; 33 Departmento de Patologia, Hospital Universitario Austral, Pilar, Argentina; 34 Department of Pathology, Hospital de Oncología Maria Curie, Buenos Aires, Argentina; 35 Department of Pathology, Sanatorio Mater Dei, Buenos Aires, Argentina; 36 Department of Research, Instituto Nacional de Enfermedades Neoplasicas, Lima, 15038 Peru; 37 Institute of Biological Sciences, Mohammed VI Polytechnic University (UM6P), 43 150 Ben-Guerir, Morocco; 38 Department of Pathology, Faculty of Medicine, UKM Medical Centre, Kuala Lumpur, Malaysia; 39 Department of Pathology, Fudan University Cancer Center, Shanghai, China; 40 Kenyatta National Hospital, Nairobi, Kenya; 41 Praava Health, Dhaka, Bangladesh; 42 Molecular Immunology Unit, Institut Jules Bordet, Université Libre de Bruxelles, Brussels, Belgium; 43 Laboratory of Tumour Immunology and Immunotherapy, Department of Oncology, KU Leuven, Leuven, Belgium; 44 Department of Hematology/Oncology, Mayo Clinic, Jacksonville, FL USA; 45 Department of Histopathology, Cambridge University Hospitals NHS Foundation Trust, Cambridge, UK; 46 Breast Cancer Program, Vanderbilt-Ingram Cancer Center, Vanderbilt University Medical Center, Nashville, TN USA; 47 Clinical Oncology Unit, Instituto Oncológico Córdoba, Córdoba, Argentina; 48 India Cancer Research Consortium-ICMR, Department of Health Research, New Delhi, India; 49 Department of Pathology, Laboratorio QUANTUM, Rosario, Argentina; 50 Department of Clinical Genetics and Pathology, Skåne University Hospital, Lund University, Lund, Sweden; 51 Department of Pathology, Yale School of Medicine, New Haven, CT USA; 52 Diagnostic Development, Ontario Institute for Cancer Research, Toronto, Canada; 53 Cancer Research UK Edinburgh Centre, Institute of Genetics and Cancer, The University of Edinburgh, Edinburgh, UK; 54 Department of Laboratory Medicine and Pathobiology, University of Toronto, Toronto, Canada; 55 Department of Pathology, Istituto Europeo di Oncologia IRCCS, and University of Milan, Milan, Italy; 56 Institute of Pathology, Universitätsklinikum Gießen und Marburg GmbH, Standort Marburg and Philipps-Universität Marburg, Marburg, Germany; 57 Department of Pathology, Matsuyama Shimin Hospital, Matsuyama, Japan; 58 Department of Medical Oncology, Institut Jules Bordet, Université Libre de Bruxelles, Brussels, Belgium; 59 German Breast Group, Neu-Isenburg, Germany; 60 Laboratory of Pathology, National Cancer Institute, NIH, Bethesda, MD USA; 61 Department of Pathology and Laboratory Medicine, Indiana University, Indianapolis, USA; 62 Department of Pathology, The University of Texas M.D. Anderson Cancer Center, Houston, TX USA; 63 National Surgical Adjuvant Breast and Bowel Project (NSABP)/NRG Oncology, Pittsburgh, PA USA; 64 Department of Pathology, RCCS Fondazione Istituto Nazionale Tumori and University of Milan, School of Medicine, Milan, Italy; 65 Sir Peter MacCallum Department of Oncology, University of Melbourne, Parkville, VIC Australia; 66 Medical Oncology Department, Peter MacCallum Cancer Centre, Melbourne, Australia; 67 Department of pathology Sanatorio Mater Dei, Buenos Aires, Argentina; 68 Department of Pathology and Laboratory Medicine, Kurume University Medical Center, Kurume, Fukuoka, Japan; 69 Servicio de Anatomía Patológica, Hospital Italiano de Buenos Aires, Buenos Aires, Argentina; 70 Division of Pathology, The Netherlands Cancer Institute, Amsterdam, The Netherlands; 71 Department of Pathology, GZA-ZNA Hospitals, Antwerp, Belgium; 72 Perlmutter Cancer Center, New York University Medical School, New York, NY USA; 73 Divisions of Medical Oncology, Molecular Oncology & Immunology, The Netherlands Cancer Institute, Amsterdam, The Netherlands; 74 Department of Surgery, Oncology and Gastroenterology, University of Padova, Padova, Italy; 75 Medical Oncology 2, Istituto Oncologico Veneto IOV-IRCCS, Padova, Italy; 76 Service de Biostatistique et d’Epidémiologie, Gustave Roussy, Oncostat U1018, Inserm, University Paris-Saclay, labeled Ligue Contre le Cancer, Villejuif, France; 77 Department of Radiation Oncology, Department of Pathology and Laboratory Medicine, Weill Cornell Medicine, New York, NY USA; 78 Department of Pathology, AZ Sint-Maarten, Mechelen, Belgium; 79 Department of Pathology, VCU, Richmond, VA USA; 80 Pathomorphology, Buzoo KOD, Omsk, Russia; 81 Anatomical Pathology, HUFA, Alcorcón, Spain; 82 Pathology, Hospital Clinic Barcelona, Barcelona, Spain; 83 Pathology, The Catholic University of Korea, Seoul, South Korea; 84 Breast Surgery, the First Hospital of Jiaxing, Jiaxing, China; 85 Oncology, General Teaching hospital Prague, Prague, Czech Republic; 86 Medical Oncology, Centro Oncologico De Galicia, A Coruña, Spain; 87 Pathology, Istanbul University-Cerrahpasa, Medical School, Istanbul, Turkey; 88 Pathology, Tata Memorial Hospital, ACTREC, Mumbai, India; 89 Pathology, Hospital israelita Albert Einstein, São Paulo, Brazil; 90 Histopathology, Sullivan Nicolaides, Darwin City, NT Australia; 91 Anatomical Pathology, Singapore General Hospital, Singapore, Singapore; 92 Dentistry, Universidade Estadual de Montes Claros (UNIMONTES), Montes Claros, Brazil; 93 Anatomical Pathology, Faculdade de Medicina do ABC, Santo André, Brazil; 94 Institut für Pathologie, Klinikum Bayreuth, Bayreuth, Germany; 95 Department of Pathology, Mater Misericordiae University Hospital, Dublin, Ireland; 96 Department of Pathology, ULSS 6 Euganea, Padua, Italy; 97 Department of Oncology/Pathology, Karolinska Insitutet, Solna, Sweden; 98 Breast surgery, Memorial Sloan Kettering Cancer Center, Boston, MA USA; 99 Department of Pathology, University of Ulsan College of Medicine, Asan Medical Center, Ulsan, Korea; 100 Pathology, Complejo hospitalario universitario insular materno infantile, Las Palmas de Gran Canaria, Las Palmas, Spain; 101 Medical Oncology, Kyungpook National University Hospital, Daegu, South Korea; 102 Department of Pathology, Mexican Oncology Hospital, IMSS, Mexico, Mexico; 103 Medical Oncology, Clínica AMO, Salvador, Brazil; 104 Pathology, Complex oncology center Plovdiv, Plovdiv, Bulgaria; 105 Anatomical Pathology, The Oncology Institute Cluj-Napoca, Cluj-Napoca, Romania; 106 Pathology, Imagepat, Porto Alegre, Brazil; 107 Medical Oncology, Azienda Ospedaliera Regionale San Carlo, San Carlo, Italy; 108 Pathology, Virginia Commonwealth University Health, Virgina, VA USA; 109 Pathology, AIIMS, Bhopal, India; 110 Pathology, Katedra Patomorfologii UJ CM, Kraków, Poland; 111 Pathology, Unipath, Jaipur, India; 112 Department of Pathology, Medical Faculty of Porto University, Porto, Portugal; 113 Pathology, Hospital das Clínicas da Faculdade de Medicina da Universidade de São Paulo, São Paulo, Brazil; 114 Department of Pathology, Keio University School of Medicine, Tokyo, Japan; 115 Pathology, Unidad de Patologia y Reumatologua, Mexico, Mexico; 116 Pathology, Hospital Universitario de Canarias, Tenerife, Spain; 117 Pathology, IRCCS sacrocuore don calabria, Verona, Italy; 118 U.O. Anatomia Patologica, ASST Lariana-Ospedale “Sant’Anna di Como”, Vicenza, Italy; 119 Laboratory Medicine, De La Salle University Medical Center, De La Salle, Philippines; 120 Pathology, Instituto Nacional de Cancerologia, Colombia, USA; 121 Pathology, Liverpool Clinical Laboratories, Liverpool University Hospitals NHS Trust, Liverpool, UK; 122 Pathology, NSW Health, Gosford, NSW Australia; 123 Life Sciences, Ahmedabad University, Gujarat, India; 124 Pathology, Tata Memorial Centre, Mumbai, India; 125 Faculté de Médecine, Université Mouloud MAMMERI, Tizi Ouzou, Algeria; 126 Pathology, Hung Vuong Hospital, Ho Chin Minh, Hanoi, Vietnam; 127 Driving, Imperial Driving School, Kisumu City, Kenya; 128 Iovance Biotherapeutics, San Carlos, CA USA; 129 Breast Surgery, Nakagami Hospital, Okinawa, Japan; 130 Pathology, Q2 Solutions, Bathgate, UK; 131 NSW Health Pathology, St George Hospital, Sydney, NSW Australia; 132 Department of Pathology and Laboratory Medicine, Emory University, Atlanta, GA USA; 133 Histogenex, Beijing, China; 134 Department of Pathology, NSW Health Pathology, Sydney, NSW Australia; 135 ASL Napoli 1 Centro, Ospedale Pellegrini, Napoli, Italy; 136 Anatomic Pathology for Clinical Trials, Quest Diagnostics, California, USA; 137 Department of Pathology, Argos - Fortaleza-CE, Fortaleza, Brazil; 138 Anatomic Pathology, Q2 Solutions, California, USA; 139 Pathology, Q2 lab solutions and Henry Mayo Newhall Hospital, California, USA; 140 UOC Anatomia Patologica, ULSS5 Polesana Rovigo, Rovigo, Italy; 141 Pathology, DPMG, Sacramento, USA; 142 Anatomic Pathology, Q2 Lab Solutions, San Juan Capistrano, California USA; 143 Anatomic Pathology, Q2 Lab Solutions, Bathgate, UK; 144 Department of Oncology, IMIP, Northeast, Brazil; 145 Surgical Pathology, San Cecilio Hospital, Granada, Spain; 146 Anatomical Pathology, NSW Health, Sydney, Australia; 147 Histopathology Department, Betsi Cadwaladr University Health Board, North Wales, UK; 148 Pathology, Federal University of Pernambuco, Recife, Brazil; 149 School of Control Science and Engineering, Shandong University, Jinan, China; 150 Pathology, Gustave Roussy Institut, Villejuif, France; 151 Anatomical Pathology, Royal North Shore Hospital, St Leonards, NSW Australia; 152 Department of Pathology, Stavanger University Hospital, Stavanger, Norway; 153 Q2 Solutions, Atlanta, GA USA; 154 Pathology, Krasnoyarsk State Medical University, Krasnoyarsk, Russia; 155 Pathological Anatomy, Padova University Hospital, Padua, Italy; 156 Division of Imaging, Diagnostics, and Software Reliability, US Food and Drug Administration, Silver Spring, MD USA; 157 School of Medicine, Medical Science and Nutrition, University of Aberdeen, Aberdeen, Scotland; 158 Multidisciplinary Oncologic Centre Antwerp (MOCA), Antwerp University Hospital, Edegem, Belgium; 159 Public Health and Primary Care, Katholieke Universiteit Leuven, Leuven, Belgium; 160 Pathology, Washington University at St. Louis, St. Louis, MO USA; 161 Optimisation and Pattern Recognition Group, University of Melbourne, Melbourne, Australia; 162 Anatomic Pathology, ASST Bergamo Ovest, Treviglio, Italy; 163 Pathology Department, Hospital Quiron Salud, Madrid, Spain; 164 Department of Pathology, University of Pennsylvania, Philadelphia, USA; 165 Department of Pathology, Nakagami Hospital, Okinawa, Japan; 166 Department of Pathology, Erasmus MC, Cancer Institute, Rotterdam, The Netherlands; 167 Medical Oncology, San Raffaele Scientific Institute, Milan, Italy; 168 Anatomic Pathology, Istanbul Florence Nightingale Hospital, Ýstanbul, Turkey; 169 Cancer Biology and Therapeutics Laboratory, School of Biomolecular and Biomedical Science, UCD Conway Institute, Dublin, Ireland; 170 Precision Oncology Ireland, Conway Institute, UCD, Dublin, Ireland; 171 Ohio State University Comprehensive Cancer Center, Colombus, OH USA; 172 Medical Oncology, Centro Hospitalar Universitario do Algarve, Faro, Portugal; 173 Medical Biophysics, University of Toronto, Toronto, Canada; 174 Histogenex, Antwerpen, Belgium; 175 Institute of Pathology Heidelberg (IPH), University Hospital Heidelberg, Heidelberg, Germany; 176 Head breast Cancer Program, Oncology Department, IOB institute of Oncology, Quiron Group, Barcelona, Spain; 177 Department of Pathology, University Hospital, Sofia, Bulgaria; 178 Public Health and Primary Care, KU Leuven, Leuven, Belgium; 179 Department of Oncology, Hospital de Mataro, Barcelona, Spain; 180 Department of Pathology, University of Kansas Medical Center, KansasCity, KS USA; 181 Tisch Cancer Institute, Icahn School of Medicine at Mount Sinai, New York, NY USA; 182 Adelson School of Medicine, Ariel University, Ramat Gan, Israel; 183 Institute of Cancer Research, London, UK; 184 Memorial Sloan Kettering Cancer Centre, Department of Pathology, New York, NY USA; 185 Department of Pathology, Léon Bérard Cancer Center, Lyon, France; 186 Department of Pathology, Saint-Petersburg clinical scientific and practical center for specialised types of medical care (oncological), St.Petersburg, Russia; 187 Fundacion Instituto Valenciano de Oncologia, Valencia, Spain; 188 Histopathology, Aster Labs, Shoreview, St Paul, MN USA; 189 St. Vincent’s Hospital, The Catholic University of Korea, Seoul, South Korea; 190 Department of Pathology, University of Marburg, Marburg, Germany; 191 Department of Oral and Maxillofacial Diseases, Helsinki, Finland; 192 Faculty of Medicine and Health Sciences, Macquarie University, Sydney, Australia; 193 KU Leuven- University of Leuven, Department of Imaging and Pathology, Laboratory of Translational Cell & Tissue Research and KU Leuven- University Hospitals Leuven, Department of Pathology, Leuven, Belgium; 194 Institut für Pathologie, UK Hamburg, Hamburg, Germany; 195 Department of Surgical Pathology, JR Kyushu Hospital, Kitakyushu, Fukuoka, Japan; 196 Biomedical Quality Assurance Research Unit, KU Leuven, Leuven, Belgium; 197 Cancer Bioinformatics Lab, Cancer Centre at Guy’s Hospital, London, UK; 198 School of Life Sciences and Medicine, King’s College London, London, UK; 199 Department of Veterinary Medicine, University of Perugia, Perugia, Italy; 200 Department of Pathology, Karolinska Institute, Solna, Sweden; 201 Consulting, PixelMed, Bangor, PA USA; 202 Department of Pathology and Laboratory Medicine, UNC School of Medicine, Chapel Hill, NC USA; 203 Department of Laboratory Medicine and Molecular Diagnostics, Sunnybrook Health Sciences Centre, Toronto, Canada; 204 Roche Diagnostics, Brussel, Belgium; 205 Department of Radiation Oncology, Sunnybrook Health Sciences Centre, Toronto, ON Canada; 206 Department of Radiation Oncology, University of Toronto, Toronto, Canada; 207 Breast Cancer Now Research Centre, The Institute of Cancer Research, London, UK; 208 Merck & Co., Inc, Kenilworth, NJ USA; 209 Oncology Merck & Co, Kenilworth, NJ USA; 210 Pathology Department, Hospital General Universitario Gregorio Marañón, Madrid, Spain; 211 Catalan Institute of Oncology, Hospital Germans Trias i Pujol, Badalona, Spain; 212 Pathology and Molecular Diagnostics department, MD Anderson Cancer Center Madrid, Madrid, Spain; 213 Roche, Tucson, AZ USA; 214 Department of Biomedical Informatics and Department of Pathology, Stony Brook School of Medicine, Stony Brook, NY USA; 215 Comprehensive Cancer Center, Fondazione Policlinico Universitario Agostino Gemelli IRCCS, Università Cattolica del Sacro Cuore, Roma, Italy; 216 Medical Oncology Unit, Agostino Gemelli IRCCS Polyclinic Foundation, Rome, Italy; 217 Department of Oncology and Pathology, Karolinska Institutet, Stockholm, Sweden; 218 Departments of Epigenetics and Molecular Carcinogenesis, The University of Texas MD Anderson Cancer Center, Houston, TX USA; 219 Department of Clinical Pathology, Akademiska University Hospital, Uppsala, Sweden; 220 Department of pathology, Maastricht University Medical Centre, Maastricht, The Netherlands; 221 GHI Le Raincy-Montfermeil, Chelles, Île-de-France, Montfermeil, France; 222 Department of Medical Biology and Pathology, Gustave Roussy Cancer Campus, Villejuif, France; 223 Laboratory of Molecular and Tumor Immunology, Earle A. Chiles Research Institute/ Providence Cancer Center, Portland, Oregon USA; 224 Department of Pathology, Breast Pathology Section, Northwestern University, Chicago, IL USA; 225 Harry Perkins Institute of Medical Research, University of Western Australia, Crawley, WA Australia; 226 Department of Laboratory Medicine and Pathology, University of Minnesota, Minneapolis, MN USA; 227 Departamento de Anatomia Patologica, Hospital de Terrassa, Barcelona, Spain; 228 Department of Pathology, Netherlands Cancer Institute, Amsterdam, The Netherlands; 229 Department of Anatomic Pathology, Faculty of Medicine, Universidad De La Frontera, Temuco, Chile; 230 Department of Pathology, Universidad de La Frontera, Temuco, Chile; 231 Department of Pathology and Laboratory Medicine, Tufts Medical Center, Boston, USA; 232 Institute of Cancer Research Clinical Trials and Statistics Unit, The Institute of Cancer Research, Surrey, UK; 233 Department of Medical Sciences, University of Turin, Turin, Italy; 234 The Fingerland Department of Pathology, University Hospital Hradec Kralove, Kralove, Czech Republic; 235 Department of Surgical Pathology Zealand University Hospital, Zealand, Denmark; 236 Department of Pathology, Semmelweis University, Budapest, Hungary; 237 Department of Pathology, St Vincentfs University Hospital and University College Dublin, Dublin, Ireland; 238 University of Turin / Candiolo Cancer Institute - FPO, IRCCS, Candiolo, Italy; 239 Servico de Anatomia Patologica, Centro Hospitalar Universitario de Sao Joao and Ipatimup, Porto, Portugal; 240 University of Turin at Candiolo Cancer Institute, FPO-IRCCS, Candiolo, Italy; 241 Nuffield Department of Population Health, University of Oxford, Oxford, UK; 242 Department of Medical Oncology, University Hospitals Bristol NHS Foundation Trust, Bristol, UK; 243 Institut Curie, Department of Pathology, Inserm U934,Paris Sciences Lettres University, Paris, France; 244 Pharmaceutical Research and Early Development (pRED), Roche Innovation Center Munich, Penzberg, Germany; 245 Department of Surgical Pathology, gSaint Savvash Regional Anticancer Hospital, Athens, Greece; 246 Department of Pathology, Herlev and Gentofte University Hospital, Herlev, Denmark; 247 Dipartimento di Scienze della Salute (DSS), Firenze, Italy; 248 Department of Pathology, Medical University of Vienna, Vienna, Austria; 249 Department of Physics and Astronomy, University of California, Irvine, Irvine, CA USA; 250 Diagnostic and Research Institute of Pathology, Medical University of Graz, Graz, Austria; 251 The Gillies McIndoe Research Institute, Wellington, New Zealand; 252 Department of Pathology, BC Cancer Agency Vancouver Centre, Department of Pathology, Vancouver, Canada; 253 Department of Pathology, University Hospital, Geneva, Switzerland; 254 Division of Imaging, Diagnostics, and Software Reliability, Office of Science and Engineering Laboratories, Center for Devices and Radiological Health, US Food and Drug Administration, Silver Spring, MD USA; 255 University Hospital Halle (Saale), Institute of Pathology, Halle (Saale), Germany; 256 Oncology Department, Cancer Institute, University College London, London, UK; 257 Department of Pathology, University Hospital of Bellvitge, Oncobell, IDIBELL, LfHospitalet del Llobregat, Barcelona, 08908 Catalonia Spain; 258 Department of molecular pathology, Netherlands Cancer Institute, Amsterdam, The Netherlands; 259 Translational Research, Forbius, Montreal, Canada; 260 Vall dfHebron Hospital Research Institute, Autoimmune Diseases department, Barcelona, Spain; 261 Research Pathology, Genentech Inc., South San Francisco, CA USA; 262 Department of Pathology, GZA-ZNA Ziekenhuizen, Wilrijk, Belgium; 263 Translational Gerontology Branch, National Institute on Aging, National Institutes of Health, Baltimore, MD 21224 USA; 264 Experimental Gerontology Section and Translational Gerontology Branch, NIA, NIH, Baltimore, USA; 265 Masaryk Memorial Cancer Institute, Brno, Czech Republic; 266 Department of Pathology and Cell Biology, Faculty of Medicine, Montreal University, Montreal, Canada; 267 Scientific Director, Image Analysis, AstraZeneca Computational Pathology, Munich Area, Germany; 268 Pathology, CHU UCL, Namur, Belgium; 269 Anatomical Pathology, Hospital Italiano de Buenos Aires, Buenos Aires, Argentina; 270 Anatomical Pathology, Austin Health, Heidelberg, Australia; 271 University of Sydney, Sydney, Australia; 272 Department of Medical Oncology, Peter MacCallum Cancer Centre, Melbourne, Australia; 273 Department of Medicine, University of Melbourne, Parkville, Australia; 274 Directorate of Surgical Pathology, SA Pathology, Adelaide, Australia; 275 Consumer Advisory Panel, Breast Cancer Trials, Adelaide, Australia; 276 Department of Anatomical Pathology, Royal Melbourne Hospital, Parkville, Australia; 277 The Cancer Research Program, Garvan Institute of Medical Research, Darlinghurst, Australian Clinical Labs, Darlinghurst, Australia; 278 Department of Pathology, Peter MacCallum Cancer Centre, Melbourne, Australia; 279 Division of Research and Cancer Medicine, Peter MacCallum Cancer Centre, University of Melbourne, Victoria, Australia; 280 The University of Queensland Centre for Clinical Research and Pathology Queensland, Brisbane, Australia; 281 NSW Health Pathology, Sydney, Australia; 282 Department of Medical Oncology, Austin Health, Heidelberg, Australia; 283 Institute of Pathology, Medical University of Graz, Graz, Austria; 284 Department of Medicine, Clinical Division of Oncology, Comprehensive Cancer Centre Vienna, Medical University of Vienna, Vienna, Austria; 285 Department of Pathology, Hospital Graz II, Academic Teaching Hospital of the Medical University Graz, Graz, Austria; 286 Department of Radiation Oncology, Iridium Kankernetwerk, Wilrijk- Antwerp, Belgium; 287 Faculty of Medicine and Health Sciences, University of Antwerp, Wilrijk-Antwerp, 2610 Antwerpen, Belgium; 288 Department of Radiology, Jules Bordet Institute, Bruxelles, Belgium; 289 Department of Pathology, AZ Turnhout, Turnhout, Belgium; 290 Department of Pathology, Jules Bordet Institute, Brussels, Belgium; 291 HistoGeneX NV, Antwerp, Belgium and AZ Sint-Maarten Hospital, Mechelen, Belgium; 292 Department of Pathology, University Hospital Antwerp, Edegem, Belgium; 293 Department of Pathology, University Hospital Ghent, Gent, Belgium; 294 Medical Oncology, GZA, Antwerp, Belgium; 295 Pathology department of ZNA and GZA hospitals in Antwerp, Antwerp, Belgium; 296 Institut Jules Bordet, Universite Libre de Bruxelles, Brussels, Belgium; 297 Department of Pathology, Cliniques Universitaires Saint-Luc, Brussels, Belgium; 298 Department of Pathology, Institut Jules Bordet, Universite Libre de Bruxelles, Brussels, Belgium; 299 Department of Pathology, Institut Jules Bordet, Brussels, Belgium; 300 Department of Development and Regeneration, Laboratory of Experimental Urology, KU Leuven, Leuven, Belgium; 301 Bioinformatics and Computational Genomics Laboratory, Princess Margaret Cancer Center, Toronto, Canada; 302 Trev & Joyce Deeley Research Centre, British Columbia Cancer Agency, Victoria, Canada; 303 Department of Pathology and Laboratory Medicine, University of British Columbia, Vancouver, Canada; 304 University of British Columbia, Vancouver, British Columbia Canada; 305 Genetic Pathology Evaluation Centre, Department of Pathology and Laboratory Medicine, University of British Columbia, Vancouver, Canada; 306 Center for Pharmacogenomics and Fudan-Zhangjiang, Center for Clinical Genomics School of Life Sciences and Shanghai Cancer Center, Fudan University, Shanghai, China; 307 Department of Pathology, Herlev and Gentofte Hospital, Herlev, Denmark; 308 DTU Compute, Department of Applied Mathematics, Technical University of Denmark; Visiopharm A/S, Horsholm, Denmark; 309 Department of Pathology, University of Helsinki and University of Turku, Turku, Finland; 310 Department of Otorhinolaryngology - Head and Neck Surgery, University of Helsinki and Helsinki University Hospital, Helsinki, Finland; 311 Helsinki University Central Hospital, Helsinki, Finland; 312 Institute for Molecular Medicine Finland, University of Helsinki, Helsinki, Finland; 313 Gustave Roussy, Universite Paris-Saclay, Villejuif, France; 314 Université Paris-Sud, Institut National de la Santé et de la Recherche Médicale, Villejuif, France; 315 Université Paris-Saclay, Univ. Paris-Sud, Villejuif, France; 316 Service de biostatistique et dfepidemiologie, Gustave Roussy, Villejuif, France; 317 Department of Medical Oncology, Gustave Roussy, Villejuif, France; 318 Centre de Lutte Contre le cancer - Centre Jean Perrin, Clermont-Ferrand, France; 319 R&D UniCancer, Paris, France; 320 Department of Pathology, Gustave Roussy, Grand Paris, France; 321 Department of Pathology, Gustave Roussy, Villejuif, France; 322 Service de Biostatistique et d’Epidémiologie, Gustave Roussy, CESP, Université-Paris Sud, Université Paris-Saclay, Villejuif, France; 323 Department of Surgical Pathology and Biopathology, Jean Perrin Comprehensive Cancer Centre, Clermont-Ferrand, France; 324 Institute of Pathology, Charite Universitätsmedizin Berlin, Berlin, Germany; 325 Breast Center, Dept. OB&GYN and CCC (LMU), University of Munich, Munich, Germany; 326 Johanniter GmbH - Evangelisches Krankenhaus Bethesda Mönchengladbach, West German Study Group, Monchengladbach, Germany; 327 Charité - Universitätsmedizin Berlin, corporate member of Freie Universität Berlin, Humboldt-Universität zu Berlin, Berlin, Germany; 328 Berlin Institute of Health, Institute of Pathology, Charitéplatz 1, 10117 Berlin, Germany; 329 Department of Pathology, Bács-Kiskun County Teaching Hospital, Kecskemét, Hungary; 330 Department of Pathology, University of Szeged, Szeged, Hungary; 331 Department of Pathology, Istituto Europeo di Oncologia, University of Milan, Milan, Italy; 332 Department of Medical Oncology, Istituto Europeo di Oncologia, Milan, Italy; 333 Azienda AUSL, Regional Hospital of Aosta, Aosta, Italy; 334 University of Milano, Istituto Europeo di Oncologia, IRCCS, Milano, Italy; 335 Departments of Surgical Pathology, Kyoto Prefectural University of Medicine, Kyoto, Japan; 336 Kyoto University, Department of Breast Surgery, Kyoto, Japan; 337 Kansai Medical University Medical Center, Osaka, Japan; 338 Department of Breast Surgery, Kyoto University, Kyoto, Japan; 339 Department of Pathology and Laboratory Medicine, Kansai Medical University, Osaka, Japan; 340 Breast Surgery, Kansai Medical University Hospital, Osaka, Japan; 341 Medical Oncology, Fukushima Medical University Hospital, Fukushima, Japan; 342 Japanese Foundation for Cancer Research, Tokyo, Japan; 343 Department of Diagnostic Pathology, Nippon Medical School Hospital, Tokyo, Japan; 344 Chugai Pharmaceutical Co., Ltd, Tokyo, Japan; 345 Department of Pathology, Kawasaki Medical School, Okayama, Japan; 346 Department of Diagnostic Pathology, Kyoto University, Kyoto, Japan; 347 National Cancer Center Hospital, Tokyo, Japan; 348 Department of Surgery, Kansai Medical School, Hirakata, Japan; 349 Department of Pathology, Mie University School of Medicine, Mie, Japan; 350 Pathology, Aga Khan University Hospital, Nairobi, Kenya; 351 Department of Pathology and Diagnostic Laboratory Services, , UKM Medical Centre, Kuala Lumpur, Malaysia; 352 Surgical Pathologist, North Shore Hospital, WDHB, Auckland, New Zealand; 353 Department of Oncology & Radiotherapy, Medical University of Gda.sk, Gdansk, Poland; 354 Tumor Pathology Department, Maria Sklodowska-Curie Memorial Cancer Center, Gliwice, Poland; 355 Breast Unit, Champalimaud Clinical Center/Champalimaud Foundation, Lisbon, Portugal; 356 Breast Unit, Champalimaud Clinical Centre, Lisboa, Portugal; 357 Department of Oncology, National Cancer Centre, Singapore, Singapore; 358 Division of Medical Oncology, National Cancer Centre Singapore, Singapore, Singapore; 359 Medical Oncology Service, Hospital General Universitario Gregorio Maranon, Universidad Complutense, Madrid, Spain; 360 Palex Medical SA - Exact Sciences Corp, Madrid, Spain; 361 Department of Oncology, IVO Valencia, Valencia, Spain; 362 Pathology Department, Instituto de Investigacion Sanitaria Fundación Jimenez Díaz (IIS-FJD), Madrid, Spain; 363 GEICAM-Spanish Breast Cancer Research Group, Madrid, Spain; 364 Pathology Department, Hospital del Mar, Parc de Salut Mar, Barcelona, Spain; 365 Molecular Oncology Group, Vall dfHebron Institute of Oncology, Barcelona, Spain; 366 Department of Pathology, Hospital General Universitario de Castelló, Castello, Spain; 367 Pathology Department, H.U. Vall dfHebron, Barcelona, Spain; 368 Medical Oncology, The Netherland Cancer Institute, Amsterdam, The Netherlands; 369 Computational Pathology Group, Department of Pathology, Radboud University Medical Center, Nijmegen, The Netherlands; 370 Division of Molecular Pathology, The Netherlands Cancer Institute, Amsterdam, The Netherlands; 371 Department of Research IT, The Netherlands Cancer Institute, Amsterdam, The Netherlands; 372 Department of Pathology, The Netherlands Cancer Institute, Amsterdam, The Netherlands; 373 Division of Molecular Oncology & Immunology, The Netherlands Cancer Institute, Amsterdam, The Netherlands; 374 Department of Pathology, Academic Medical Center, Amsterdam, The Netherlands; 375 Medical Oncology, Netherlands Cancer Institute, Amsterdam, The Netherlands; 376 Medical Oncology, University Hospital Southampton NHS Foundation Trust, Southampton, UK; 377 Translational Breast Radiobiology, Institute of Cancer Research, Honorary Consultant Clinical Oncologist (Breast), The Royal Marsden, London, UK; 378 Breast Cancer Now Toby Robins Research Centre, The Institute of Cancer Research, London, UK; 379 Cancer Research UK Lung Cancer Centre of Excellence, University College London Cancer Institute, University College London, London, UK; 380 Department of Pathology, UCL Cancer Institute, UCL, London, UK; 381 University College Hospitals NHS Trust, London, UK; 382 Vivactiv Ltd, Bellingdon, Bucks, UK; 383 Leicester Cancer Research Centre, University of Leicester, Leicester, and MRC Toxicology Unit, University of Cambridge, Cambridge, UK; 384 Centre for Evolution and Cancer; Division of Molecular Pathology, The Institute of Cancer Research, London, UK; 385 Clinical Trial Service Unit & Epidemiological Studies Unit, University of Oxford, Oxford, UK; 386 Guy’s Hospital, London, UK; 387 King’s College London, London, UK; 388 Department of Pathology, Wexner Medical Center at the Ohio State University, Columbus, OH USA; 389 Mayo Clinic, Rochester, Minnesota Joint Appointment, Department of laboratory Medicine and Pathology, Research Chair, Division of Medical Oncology, Robert Mutter Radiation Oncology, Mayo Clinic, Rochester, MN USA; 390 Albert Einstein College of Medicine in New York, New York, NY USA; 391 Division of Anatomic Pathology, University of California San Diego Health System, San Diego, CA USA; 392 Surgical Oncology, Baylor College of Medicine, Houston, TX USA; 393 Departments of Pathology, Genomic Medicine, Dermatology, and Translational Molecular Pathology, The University of Texas MD Anderson Cancer Center, Houston, TX USA; 394 PhenoPath Laboratories, Seattle, WA USA; 395 Department of Medical Oncology, Dana-Farber Cancer Institute, Boston, MA USA; 396 Case Western Reserve University; Louis Stokes Cleveland Veterans Health Administration Medical Center, Cleveland, OH USA; 397 Pulmonary Pathology, New York University Center for Biospecimen Research and Development, New York University, New York, NY USA; 398 Department of Pathology, Johns Hopkins Hospital, Baltimore, MD USA; 399 PathAI, Inc., Cambridge, MA USA; 400 Department of Pathology, University of Iowa Hospitals and Clinics, Iowa City, IA USA; 401 Breast Cancer Trials, Women’s Malignancies Disease Group, The Johns Hopkins Kimmel Cancer Center, Baltimore, MD USA; 402 Department of Pathology, University Hospitals Cleveland Medical Center affiliated with Case Western University, Cleveland, OH USA; 403 Department of Biomedical Informatics, Emory University, Atlanta, GA USA; 404 Departments of Pathology and Oncology, The Johns Hopkins Hospital, Baltimore, MD USA; 405 National Surgical Adjuvant Breast and Bowel Project Operations Center/NRG Oncology, Pittsburgh, PA USA; 406 Department of Pathology, New York University Langone Medical Centre, New York, NY USA; 407 Department of Computational and Systems Biology, University of Pittsburgh, Pittsburgh, PA USA; 408 Mayo Clinic Breast SPORE, Mayo Clinic, Rochester, MN USA; 409 Department of Pathology, Brigham and Women’s Hospital, Boston, MA USA; 410 Dana-Farber Cancer Institute, Boston, MA USA; 411 Oncology Clinical Development, Bristol-Myers Squibb, Princeton, NJ USA; 412 Department of Medicine, Vanderbilt University Medical Centre, Nashville, TN USA; 413 Department of Cancer Biology, Mayo Clinic, Jacksonville, FL USA; 414 Human Oncology and Pathogenesis Program, Memorial Sloan Kettering Cancer Center, New York, NY USA; 415 Department of Pathology, Dana Farber Cancer Institute, Boston, MA USA; 416 National Cancer Institute, Bethesda, MD USA; 417 Division of Medical Oncology, Department of Medicine, University of Colorado Anschutz Medical Campus, Aurora, CO USA; 418 Medical Oncology, Department of Medicine, Cedars-Sinai Medical Center, Los Angeles, CA USA; 419 PathAI Inc., Cambridge, MA USA; 420 Translational Sciences, MedImmune, Gaithersberg, MD USA; 421 Department of Pathology, Massachusetts General Hospital, Boston, MA USA; 422 Department of Laboratory Medicine and Pathology, Mayo Clinic, Rochester, NY USA; 423 Department of Medicine, Department of Obstetrics & Gynecology and Women’s Health, Albert Einstein Medical Center, Bronx, NY USA; 424 Departments of Medicine and Cancer Biology, Vanderbilt University Medical Centre, Nashville, TN USA; 425 NSABP/NRG Oncology, Pittsburgh, PA USA; 426 Yale Cancer Center Genetics, Genomics and Epigenetics Program, Yale School of Medicine, New Haven, CT USA; 427 Pathology Department, Stanford University Medical Centre, Stanford, CA USA; 428 Department of Medical Oncology, Yale University School of Medicine, New Haven, CN USA; 429 Department of Pathology, Northwestern University Feinberg School of Medicine, Chicago, IL USA; 430 Biorepository and Tissue Technology Shared Resources, University of California San Diego, San Diego, CA USA; 431 Breast Cancer Research Foundation, New York, NY USA; 432 Memorial Sloan Kettering Cancer Center, New York, NY USA; 433 Department of Oncology, Mayo Clinic, Rochester, NY USA; 434 Roche Tissue Diagnostics, Digital Pathology, Santa Clara, CA USA; 435 Department of Pathology, Microbiology and Immunology, Vanderbilt University Medical Centre, Nashville, TN USA; 436 Division of Biostatistics, Dana-Farber Cancer Institute, Boston, MA USA; 437 Harvard Medical School, Boston, MA USA; 438 Vernon Cancer Center, Newton-Wellesley Hospital, Newton, MA USA; 439 Department of Biomedical Informatics, Emory University School of Medicine, Atlanta, GA USA; 440 Division of Hematology-Oncology, Beth Israel Deaconess Medical Center, Boston, MA USA; 441 Icahn School of Medicine at Mt. Sinai, New York, NY USA; 442 Department of Pathology, Massachusetts General Hospital/Harvard Medical School, Boston, MA USA; 443 Cancer Immunotherapy Trials Network, Central Laboratory and Program in Immunology, Fred Hutchinson Cancer Research Center, Seattle, WA USA; 444 Department of Pathology, Weill-Cornell Medicine, New York, NY USA; 445 Translational Medicine, Bristol-Myers Squibb, Princeton, NJ USA; 446 Anatomic Pathology, Boston, MA USA; 447 Division of Bioinformatics and Biostatistics, U.S. Food and Drug Administration, Silver Spring, MD USA; 448 Department of Pathology and Laboratory Medicine, Rhode Island Hospital and Lifespan Medical Center, Providence, RI USA; 449 Oncology Service, Department of Medicine, Memorial Sloan Kettering Cancer Center, New York, NY USA; 450 Pathology and Tissue Analytics, Roche, Basel, Switzerland; 451 Department of Pathology, University Medical Center Utrecht, Utrecht, The Netherlands

**Keywords:** Predictive markers, Breast cancer

## Abstract

The advent of immune-checkpoint inhibitors (ICI) in modern oncology has significantly improved survival in several cancer settings. A subgroup of women with breast cancer (BC) has immunogenic infiltration of lymphocytes with expression of programmed death-ligand 1 (PD-L1). These patients may potentially benefit from ICI targeting the programmed death 1 (PD-1)/PD-L1 signaling axis. The use of tumor-infiltrating lymphocytes (TILs) as predictive and prognostic biomarkers has been under intense examination. Emerging data suggest that TILs are associated with response to both cytotoxic treatments and immunotherapy, particularly for patients with triple-negative BC. In this review from *The International Immuno-Oncology Biomarker Working Group*, we discuss (a) the biological understanding of TILs, (b) their analytical and clinical validity and efforts toward the clinical utility in BC, and (c) the current status of PD-L1 and TIL testing across different continents, including experiences from low-to-middle-income countries, incorporating also the view of a patient advocate. This information will help set the stage for future approaches to optimize the understanding and clinical utilization of TIL analysis in patients with BC.

## Introduction

The use of immune-checkpoint blockade (ICI) in clinical oncology has revolutionized patient care and improved survival outcomes in many patients with malignancies^1^. This therapeutic strategy has significantly expanded in the setting of advanced and early-stage breast cancer (BC), but much more work is needed to optimize patient selection based on tumor-based biomarkers. The presence of tumor-infiltrating lymphocytes (TILs) is believed to be predictive of response to immunotherapy, chemotherapy, and other targeted therapies^2,3^ in addition to their role as a prognostic biomarker^4,5^. Moreover, TILs in the tumor and the surrounding microenvironment are thought to reflect ongoing anti-tumor host immune response. Three main categories of the tumor microenvironment (TME) have been defined across different tumor types: immune-desert (“cold” tumors largely devoid of lymphocytes), immune-excluded (lymphocytes are present in the peritumoral stroma only), and immune-infiltrated/inflamed (“hot” tumors)^6,7^. Conceptually, each of these TME categories reflects a specific interaction between the tumor genotype/phenotype and the host immune system, which can impact the response to both conventional anticancer therapies and ICI^8^. However, there is considerable heterogeneity within each TME category, adding uncertainty to the reproducibility of the current classification of “cold” vs. “hot” vs. “intermediate” immune-subtypes. Also, there are no validated criteria to define these subtypes, either using morphology, immunostaining, transcriptomics, or their combination, limiting the impact of these descriptors in clinical trials and daily practice.

In BC, the molecular subtype of the tumor has a major influence on its interaction with the immune system. Triple-negative BC (TNBC) and HER2-positive BC are more frequently infiltrated by higher numbers of TILs than hormone receptor (HR)-positive tumors^9,10^. However, all BC subtypes include cases with TIL-infiltration. The degree of TIL infiltration has been hypothesized to reflect the tumor mutational burden (TMB), which is lower in HR-positive BC^11,12^. Higher TMB is linked to the expression of more neoantigens and has been shown to predict survival after ICI therapy in several cancer types, and recent evidence indicates this may also be the case for TNBC^13–15^. However, the correlation between mutational burden and immune composition is complex, with the degree and nature of clonality of the mutations playing a key role in determining whether they favor or hinder immune-mediated tumor control^16^. In TNBC, higher TMB and greater genomic heterogeneity have been associated with lower TILs^17^. Conceptually, this can be explained by immunoediting, which is a result of a selection of cancer cell clones with decreased immunogenicity despite the presence of many mutations^18^. This escape from immune surveillance is associated with a reduced TIL component and increased tumor clonal heterogeneity, explaining the negative association between TMB and TILs^19–21^.

In TNBC, scoring TILs in the stromal compartment (sTILs) is demonstrably reproducible and generally well-correlated with intra-epithelial TILs, with higher stromal TILs (sTILs) predicting longer survival^9,22–24^. Generally, intra-epithelial TIL density tends to be lower than stromal TIL density^23^, raising the question of whether a tumor nest-stromal barrier precludes more robust T cell infiltration^25^, and/or whether the intra-epithelial TILs have always been present (in an inactive state), as tissue-resident T-cells. CD8+ tissue-resident memory T (TRM) cells were shown to mediate BC immunosurveillance^26^. Notably, high BC infiltration by TILs contained CD8+ T lymphocytes with TRM patterns which highly express immune-checkpoint molecules^26^.

Tertiary lymphoid structures (TLS) are lymph node-like structures that arise in tissues at sites of chronic inflammation^27^. TLS has been detected in the stroma of up to 60% of BC, with the highest frequencies in HER2+ and TNBC^7,28,29^. These findings support a critical role for the stroma in shaping the TME of BC. For example, fibroblastic reticular cells, concentrated in the T cell zone of TLS, promote, maintain or suppress T cell activities via their cytokine and chemokine secretion^30^. TLS architecture is distinguished by a T cell zone adjacent to a B cell follicle, similar to secondary lymphoid organs^27^. Immune responses generated in tumor-associated TLS would thus produce immunological memory to multiple BC neoantigens and could potentially control the growth of disseminated tumor cells^31–34^. However, there are significant concerns that TLS cannot be assessed in a reproducible manner by analysis of HE-stained slides, and that B- and T-cell immunostains are needed^34^. In addition, TLS is frequently found at the tumor perimeter, often contain high endothelial venules, and when present in the tumor area are generally considered as an aggregate when quantifying TILs in BC. Their role in BC still remains to be addressed.

The importance of expanding our current understanding of the complex TME in breast and other cancers has driven the development of diverse techniques, including molecular multi-omics profiling^35^ coupled with computational deconvolution of immune cell populations^36,37^, global and single-cell transcriptomics^38,39^, and multiplex imaging^40^, to quantify TIL distribution, functional orientation and relative frequencies in individual tumor types. However, these cutting-edge investigational tools are often limited by reproducibility across studies, particularly when attempting to resolve specific immune cell subtypes. In addition, tumors with low TILs seem to pose a significant challenge to any of these techniques and platforms^41^. Machine learning techniques are in development to evaluate TIL distribution patterns and integrate the spatial information with sTILs and with molecular profiling data^42,43^. A study examining clonal heterogeneity, TMB, copy number variations, somatic mutations, and germline polymorphisms, as well as the neoantigen load for their association with immune metagene expression in the BC subtypes, did not find any distinct recurrent single gene or pathway level mutations associated with immune infiltration^21^. However, lower clonal heterogeneity was observed in TNBC and HER2+ BC associated with higher immune gene expression, which is consistent with immunoediting^21^. There is also evidence for immune escape during the in situ to invasive BC transition, with a decrease in immune activation measurable using a combination of global profiling and single-cell transcriptomics^44^. It may be hypothesized that similar immune evasion mechanisms also occur during the transition from stage I TNBC to more advanced stages of the disease. This paradigm was also recently reported in lung cancer in which immune-evasion seems to be triggered by neoantigen editing during tumor evolution^45^. In the remainder of this review, the analytical and clinical impact of sTILs in BC management will be discussed based on recent updates from human studies, particularly clinical trials.

## Updates on the analytical and clinical validity of TILs

### Challenges in establishing the analytical validity of TILs

Analytical validity is defined as how accurately a test predicts the presence or absence of a biological variable. In other words, can the “test” correctly distinguish between TILs and other immune cells?. Previous TIL inter-pathologists-reproducibility studies (RING-studies) have shown that pathologists (using an H&E slide) can reliably assess TILs with very high concordance between many pathologists on a powered number of cases and at different cut-offs^24^. There is overwhelming evidence for the clinical validity of this evaluation^46,47^. However, analytical validity cannot be formally demonstrated due to the lack of a “gold standard” against which to assess the proposed method. This may not be that important for the clinical implementation of sTILs. However, for machine learning approaches this question is crucial. Comparing two methods, for example, a pathologist vs. an automated image-analysis system provides evidence on concordance, but not accuracy^43^. All the previous TIL-RING studies have been performed with the assumption of histological accuracy^46,47^.

In order to define accuracy in the context of assessing TILs using an H&E-stained slide, it is necessary to define a “gold standard” against which to assess the proposed method, i.e., an orthogonal-type method which is used as the “gold standard”. For example, a pan-lymphocyte marker can establish the accuracy of lymphocyte identification, answering “*how often is what a pathologist calls a TIL actually a TIL?*”. This is crucial when applying machine learning methods to identify TILs^42^, since the unequivocal differentiation of a myofibroblast, an invasive lobular carcinoma (ILC) cell, and lymphocyte is not always possible on an H&E slide. Moreover, the concordance between TIL assessment by machine learning-based methods and pathologist-TIL scores is unlikely to be 100%^48^. Currently, a TIL is defined as being a lymphocyte or a plasma cell, and both are scored and defined according to classic morphological definitions. Then, we need to define a “stromal TIL” (sTIL), i.e., what is the maximal distance between a tumor cell and a TIL to define it as an sTIL. Furthermore, uncertainty amongst pathologists exists about the inclusion of stroma abutting the tumor, or all of the stroma within the total tumoral area to include intervening stroma with low/very low sTILs. Indeed, accumulating evidence suggests that sTILs touching the tumor cells may have a different molecular phenotype than sTILs distant to the main tumor bulk^25^. Kos et al.^24^ have recently reported further factors that may impact the assessment of TILs including pre- and post-analytical histology factors, particularly in the setting of retrospective analysis of trial material, and the recognition of common artifacts. In an effort to improve concordance, the *International Immuno-Oncology Biomarker Working Group, also called the TILs Working Group* (https://www.tilsinbreastcancer.org/pitfalls/) recently provided reference images and digital slides as well as accessible guidance regarding the analysis of heterogeneous immune cell infiltrates.

### The importance to assess the clinical validity of TILs in the context of clinical utility

Clinical validity refers to the presence of sufficient evidence, usually level 1 evidence of the effect of a test (biomarker) to demonstrate its validity in a clinical setting. That is, there is robust statistically validated evidence that the test (biomarker) relates to a clinical outcome (prognostic or predictive) or a specific phenotype (TILs), etc. However clinical validity alone, whilst required for changes in clinical practice, does not drive a change in practice. For this to occur, the test must have clinical utility. Clinical utility essentially requires that the test in question addresses a direct clinical need (prediction of response to therapy, prognosis, or diagnostic classification of subtypes) and *will when implemented, impact patient management*. Simply put, a test only has clinical utility when it impacts physician and patient choice on treatment or management options. The level 1 requirement for clinical validity clearly differs according to the setting^49^. However, it is clearly not optimal to assess the clinical validity of TILs without first framing the question of clinical utility correctly. Once the correct clinical question is framed, appropriate studies must be designed to generate evidence to support the clinical validity of TILs in the setting of clinical utility. In this respect, we agree with Simon et al. that prospective trials not originally designed to address tissue biomarker studies can be used to “accommodate biomarker utility” using archived samples^49^. Since only simple tools, like a microscope, are needed for the assessment of TILs, this provides tremendous opportunities to test the “clinical utility” of TILs in various settings.

### The clinical validity and utility of TILs

#### TILs in TNBC

Level 1 evidence for a biomarker^49^ can either be reached by incorporating a biomarker into a properly powered *prospective* clinical trial (level 1A) or by achieving reproducible results in *archived* tissues from independent randomized trials, designed, conducted, and analyzed as per REMARK criteria (level 1B)^50^. Using these widely accepted criteria, level 1B evidence for clinical validity of TILs as a prognostic biomarker in early-stage TNBC is well established^9,22^. Two pooled analyses of TILs, in the adjuvant setting for TNBC^22^, and in the neoadjuvant setting across BC-subtypes^9^, included studies that have evaluated TILs on archived tissue samples based on our published guidelines^23^.

In a pooled analysis (*n* = 2148), the clinical value of TILs in predicting prognosis of early-stage TNBC, including adjuvant trials of anthracycline-based chemotherapy alone or in combination with taxanes was investigated^22^. The average age of enrolled patients was 50 years, and 33% of them were lymph node-negative. The quantification of TILs showed that their average was 23%, and 77% of patients had at least 1% sTILs. Notably, sTILs were found to be significantly reduced with advanced age, larger tumor size, more positive lymph nodes, and lower histological grade. In the multivariable Cox regression model, sTILs were an independent prognostic predictor for all endpoints; each 10% increment in sTILs corresponding to an invasive disease-free survival (DFS) hazard ratio (HR) of 0.87 (95% CI: 0.83–0.91), a distant DFS HR of 0.83 (95% CI: 0.79–0.88) and an overall survival (OS) HR of 0.84 (95% CI: 0.79–0.89) (*p* < 10^−6^)^22^. Histological grade was not a prognostic factor in this study. The second pooled analysis included women with primary BC treated with neoadjuvant chemotherapy (NACT) in six randomized trials conducted by the German Breast Cancer Group^9^. It assessed the predictive value of sTILs for chemotherapy response and prognostic estimation in patients with TNBC, HER2-positive, and luminal A/B–HER2-negative BC. sTILs quantified in diagnostic core biopsies from 3771 patients were associated with pathologic complete response (pCR) after NACT across all BC subgroups. For instance, based on three predefined groups of low (0–10% immune cells in peritumoral stromal tissue), intermediate (11–59%), and high TILs (≥60%), pCR was achieved in 31% (80/260) of TNBC patients with low TILs, 31% (117/373) of TNBC patients with intermediate TILs, and 50% (136/273) of TNBC patients with high TILs (*p* < 0.0001). OS was analyzed in 2560 patients across all BC subtypes from five of the six neoadjuvant clinical trial cohorts. However, increased sTILs were associated with longer OS only in TNBC (HR: 0.92; CI: 0.86–0.99, *p* = 0.032).

Recently, Park et al.^51^ investigated the prognostic impact of sTILs in early-stage TNBC based on four multicenter cohorts (476 patients) who were *not* treated with (neo)adjuvant chemotherapy. The presence of sTILs at baseline was correlated with several clinical endpoints including OS. Multivariate analyses demonstrated that sTILs are an independent prognostic biomarker of OS (*p* = 0.015), invasive DFS and distant DFS for TNBC (*p* < 0.001 for both)^51^. A 10% increase in sTILs also positively correlated with OS (HR: 0.88; 95% CI: 0.79–0.98), invasive DFS (HR: 0.90; 95% CI: 0.82–0.97) and distant DFS (HR: 0.86; 95% CI: 0.77–0.95). In a subgroup of TNBC patients with stage I tumors and sTILs ≥ 30%, excellent 5-year survival outcomes were reached including 98% 5-year OS^51^. Indeed, the expert opinion at the 16th St. Gallen International Breast Cancer Conference has endorsed the routine reporting of sTILs in TNBC as a prognostic factor^52^ although guidelines have not yet recommended de-escalation of standard systemic therapy according to TILs. *The WHO Classification of Tumors: Breast Tumors, 5th Edition* has also endorsed histopathological TIL quantification in TNBC and HER2-positive BCs, expressed as a mean percentage of lymphoplasmacytic infiltration of the tumor stroma^53^.

The 5th Edition of the WHO classification also re-classifies medullary carcinoma, in which prominent TILs have long been recognized, into *invasive breast carcinoma of no special type with medullary pattern*^53^. These carcinomas have been characterized as showing high immune-related gene^54^ expression and it is likely that the high TILs component of these tumors contributes significantly to their favorable clinical outcomes^55,56^. The role of TILs in the histological spectrum of TNBC is yet to be fully elucidated although early data on metaplastic carcinoma suggest that TILs may have prognostic relevance^57,58^. The role of TILs in TNBC subtypes recognized as low grade and with good prognosis histological features, such as adenoid cystic carcinoma of the breast^59^, is at present unknown. The importance of histological grade in TNBC is likely to be of utility in identifying the so-called low-grade TNBC, including the rarer subtypes (for example adenoid cystic carcinoma), that have low TILs but an excellent outcome compared to the lack of clinical utility and prognostic significance of histological grading alone in TNBC NOS.

#### TILs and PD-L1 in triple-negative BC

The phase 2 double-blind placebo-controlled GeparNuevo study (NCT02685059), randomized 174 early stage TNBC patients to receive durvalumab (a PD-L1 inhibitor) combined with standard NACT and used sTILs as a biomarker for patient stratification during randomization^60^. This proof-of-concept study showed that patients with higher sTILs in both arms of the trial had significantly improved pCR rates (*p* < 0.01), although the sTILs were not specifically related to durvalumab-response. A recent translational analysis of this trial demonstrated that both continuous TMB and TILs were independent predictors of pCR^61^. Patients with high TMB had excellent pCR rates of 82% (95% CI: 60–95%) highlighting the potential of this emerging biomarker in tailoring therapy in this setting^61^. The German phase II trial (NCT03289819) compares neoadjuvant pembrolizumab combined with nab-paclitaxel vs. pembrolizumab with epirubicin and cyclophosphamide in patients with early TNBC will also investigate sTILs at baseline, in addition to other potential biomarkers such as mutational load and microbiota. In this setting, the recently released findings of IMpassion031^62^, KEYNOTE-522^63^, and I-SPY2^64^ studies suggest that response to ICIs is independent of PD-L1 status. Thus, other biomarkers of response are needed to predict outcomes in TNBC patients treated with NACT and immune-checkpoint blockade.

In metastatic or locoregionally advanced TNBC, several immunotherapy trials have demonstrated the value of immune cell infiltrate in estimating survival outcomes including phase I (NCT01375842)^65^, phase II KEYNOTE-086 (NCT02447003)^66,67^, and phase III IMpassion130 (NCT02425891)^68^ clinical trials. TILs were used to assess the activity of atezolizumab as monotherapy in a phase Ia expansion cohort in metastatic TNBC^69^. Patients’ stratification based on TILs at baseline indicated that median OS was improved in those with >10% of sTILs cut-off (12.6 vs. 6.6 months, *p* = 0.0028)^69^. In the phase III study, Schmid et al. showed that the addition of atezolizumab to nab-paclitaxel improved progression-free survival (PFS) in TNBC as compared with the addition of placebo to nab-paclitaxel, particularly in those with PD-L1 positive tumors. Median OS in this subgroup was consistently improved in both interim analyses^68,70^, but no formal statistical testing of OS in the PD-L1 positive subgroup could be performed due to the absence of OS benefit for the entire study population^70^. Hence, the FDA approval for this particular drug and assay was based on PFS, not on OS. The available tumor samples from this study were also re-assessed for the analytical concordance of various assays for PD-L1 immunohistochemistry (IHC) and the clinical utility of these assays^71^. This post-hoc analysis demonstrated that all subgroup results based on different PD-L1-based assays were suggestive of some clinical benefit (with SP142 immunopositivity apparently indicating most clinical benefit), with different HR and low concordance between the immunohistochemical assays used (SP142 vs. 22C3 vs. SP263)^71,72^. This analysis also raises confusion as to whether HR or underpowered subgroup-analyses combining different antibodies, should be used to make claims on the performance of assays or not; taking into consideration that only a biomarker-treatment interaction analysis, in the biomarker positive vs biomarker negative population and only in powered subgroup-analysis, can inform the performance of assays. It was also shown that when >20% of sTILs were present, nearly all patients had a positive PD-L1 essay, irrespective of the assay used. This indicates that sTILs are important drivers of response and that sTILs could help mitigate the well-known assay- and reproducibility issues associated with PD-L1 assessment^71,72^. In fact, numerically higher counts of sTILs were noted in TNBC patients with a positive FDA-approved SP142 assay^71^. Furthermore, in the recent biomarker-analysis of Impassion130^73^, it was shown that TILs predict benefit to atezolizumab for any PD-L1-expression.

KEYNOTE-086 enrolled 170 patients with advanced TNBC who were treated with at least one prior line of therapy and showed a 5% response rate (RR) and 20% stable disease of the subgroup with PD-L1 positive tumors (based on the 22C3 pharmDx assay) when treated with pembrolizumab monotherapy^66^. Similarly, the cohort of the trial treated with first-line pembrolizumab displayed a durable response in patients with positive-PD-L1 (21.4%; 95% CI: 13.9–31.4)^67^. These findings confirm the previously noted improved outcomes in this setting with higher TIL levels^74^. In fact, higher sTILs were associated with significantly increased ORR (OR: 1.26, 95% CI: 1.03–1.55, *p* = 0.01) and disease control rates (OR: 1.22, 95% CI: 1.02–1.46; *p* = 0.01). PD-L1 expression was also significantly correlated with the levels of sTILs (*p* < 0.001) in metastatic TNBC treated with pembrolizumab^74^.

A recent advance resulted from the KEYNOTE-119 phase III trial (NCT02555657) in which 622 previously treated metastatic TNBC patients were randomized to receive pembrolizumab as monotherapy or cytotoxic chemotherapy^13^. This study did not demonstrate significantly prolonged OS in the overall cohort nor in the PD-L1-positive subgroup (combined positive score -CPS- ≥1 or ≥10)^13^. Exploratory analysis revealed that a marked increase in the efficacy of pembrolizumab was seen when the cut-off for a combined positive score was increased to ≥20. In this study, TILs were then evaluated according to our established guidelines^23^. In the pembrolizumab arm, high distribution of TILs was observed in responders with better survival outcomes, an effect not observed in the chemotherapy arm^75^. Since sTILs were not prespecified in the trial protocol, sTILs were not considered “regulatory-proof”, despite the OS benefit demonstrated in this phase 3 setting. TILs and combined positive scores were moderately correlated and were independent predictors of outcome in patients treated with pembrolizumab. More recently, KEYNOTE-355 (NCT02819518) randomized 847 women with metastatic TNBC to receive pembrolizumab in combination with chemotherapy vs. chemotherapy plus placebo^76^. This demonstrated that patients with CPS ≥10 treated in the immunotherapy arm had improved median PFS as compared to the placebo group (9.7 vs. 5.6 months, HR: 0.65, 95% CI: 0.49–0.86; *p* = 0.0012) but not in those with CPS of 1 or more (7.6 vs. 5.6 months (HR: 0.74, 95% 0.61–0.90; one-sided *p* value not significant)^76^.

Nevertheless, biomarker analyses of TILs in such studies are all exploratory. These findings should, therefore, ideally be validated in independent prospective studies that use suitably powered phase III randomized and controlled trial design to accelerate the clinical validation of TIL-guided therapy and to provide Level 1A evidence. We strongly propose pre-specifying the use of TILs as an integral biomarker in future trial protocols. However, the FDA has recently approved pembrolizumab for adults and children with TMB-H solid tumors. Treatment efficacy was studied in a prospectively planned retrospective analysis of 10 cohorts of patients with solid malignancies with unresectable or metastatic TMB-H tumors. These patients were enrolled in a multicenter, non-randomized, open-label clinical trial (KEYNOTE-158; NCT02628067). This approval raises interesting questions, for example: is the old paradigm for obtaining level 1A evidence for biomarkers becoming obsolete or is it simply underused?. Are prospective-retrospective analyses for biomarkers that are predictive for immune therapy and indicate OS, with the level of evidence IB, and with proven analytical and clinical validity (like TILs) sufficient for regulatory approval as a predictive marker for selection of patients for immune checkpoint inhibition? Will level 1B evidence drive clinical change? Regulatory agencies approve assays and drugs, but the scientific community still must apply that knowledge in a clinical context based on a synthesis of all evidence. Is it the role of regulatory agencies to approve clinical practice changes or should this be the role of the scientific community, in a partnership with industry and the regulatory agencies?

#### TILs in ER-positive BC

Although a high percentage of sTILs in primary TNBC and HER2-positive BCs suggests a favorable prognosis, the significance in estrogen receptor (ER)-positive, HER2-negative tumors remains uncertain. This is partly because there has been less focus on ER-positive carcinomas, which are traditionally considered to demonstrate lower immunogenicity^9,77,78^. For example, the mean TILs count is significantly lower than in TNBC and HER2-positive BC^9,79,80^. The average TMB, which frequently correlates with neoantigen load, is also lower in these tumors^81^. Small studies of single-agent immune checkpoint blockade have yielded low responses in patients with pretreated metastatic ER-positive cancer^82,83^. Nevertheless, there is marked heterogeneity for TILs infiltration and mutational burden seen in ER-positive BCs, with a considerable proportion above the mean observed in triple-negative tumors^77^. The importance of TILs in ER+/HER2-ve BC can be most easily demonstrated by combining two aspects of BC; firstly, recent population-based surveys of over 350,000 BCs in the UK and USA suggest that 73% of newly diagnosed BCs are ER+/HER2−^84,85^. Given that TILs have been detected in the stroma of up to 60% of BC^7,28,29^ the majority (over half) of all BCs with TILs must therefore be in the ER+/HER2- subgroup. Put simply, the population of ER+/HER2− BCs with TILs exceeds the combined population of HER2+/TNBCs with this feature. This demonstrates the large unmet potential for exploiting immune targeted agents in ER+/HER2− patients.

Several studies have examined the impact of TIL levels on patient prognosis in primary ER-positive BC, and five of the largest studies are summarized in Table 1^9,78,86–88^. These studies retrospectively examined histological slides either from randomized controlled trials or annotated clinical cohorts. However, the material varied considerably (tissue microarray vs. core biopsy vs full face section) as did the TILs analysis methodology (H&E staining vs. IHC) and the TILs quantification statistical methods (continuous vs categorical variable). With these caveats in mind, the most notable finding from these analyses is that no study demonstrated a favorable impact of TILs on disease-free, BC-specific, or OS in ER-positive BC. In some analyses, TILs were associated with an unfavorable prognosis. For example, in a recent meta-analysis of six NACT trials, higher TILs levels were associated with a significant reduction in OS^9^. Furthermore, two studies analyzing mixed trial and/or clinical cohorts (of almost 6000 and 1400 ER-positive tumors, respectively) reported that higher levels of intratumoral CD8^+^ TILs were associated with a significant reduction in BC-specific survival^80,86^.

**Table 1:** Summary of previous studies on the prognostic value of TILs in ER+ breast cancer.

Study	Patient population	Treatment	Method of lymphocyte assessment	Impact of sTILs in prognosis	Impact of iTILs on prognosis
Loi et al.^78^	BIG 02-98 trial (*n* = 1078)	Adjuvant anthracycline chemotherapy ± taxane	Full face H&E sections—TILs assessed as a continuous variable	Each 10% increment in sTILs associated with worse OS (HR: 1.1, *p* = 0.04044) on univariate analysis	No association with DFS or OS.
Ali et al.^86^	Mixed RCT samples and clinical cohorts (*n* = 5961)	Variable (adjuvant)	IHC for CD8+ cells on tissue microarrays (TILs assessed as a categorical variable, present vs. not present)	No association with BCSS.	The presence of any iTILs is associated with worse BCSS (HR: 1.16; 95% CI: 1.02–1.32]).
					Association lost on multivariate analysis.
Dieci et al.^87^	Two RCT cohorts (*n* = 463)	Adjuvant chemotherapy vs. no chemotherapy	Full face H&E sections. TILs assessed as both continuous and categorical variables.	No association with DFS or OS.	No association with DFS or OS.
Denkert al.^9^	Meta-analysis of 6 neoadjuvant trials (*n* = 832)	Various neoadjuvant chemotherapy regimens	Core biopsies. TILs were classified as low (0–10%), intermediate (11–59%), or high (60–100%).	Intermediate TILs were associated with worse DFS (HR: 1.50, 95% CI: 1.09–2.06) and OS (HR: 2.45, 95% CI: 1.61–3.70) vs. low TILs.	N/A
				High TILs associated with worse OS (HR: 1.79, 95% CI: 1.02–3.15) vs. low TILs. No association with DFS.	
Sobral-Leite et al.^88^	Retrospective analysis of a multicenter trial (*n* = 563)	Tamoxifen or no adjuvant therapy	IHC for CD4, CD8, and FOXP3 cells	CD4 and FOXP3 lymphocytes were not significantly associated with prognosis. Patients with high CD8 cells had an increased risk of recurrence (HR = 1.98; 95% CI: 1.14–3.41)	N/A

*BCSS* breast cancer-specific survival, *DFS* disease-free survival, *IHC* immunohistochemistry, *iTILs* intratumoural TILs, *OS* overall survival, *RCT* randomized controlled trial, *sTILs* stromal TILs.

These observations must be interpreted with caution given that the molecular subtype of ER+ BC is a major confounder in all these analyses. With the introduction of PAM-50 assays in the last decade, ER-positive cancers have been divided into luminal A and luminal B tumors—the latter characterized by higher proliferation and reduced dependence on endocrine signaling^89^. Luminal A tumors have both lower TIL infiltration and TMB than luminal B tumors^79^ and luminal B cancers are associated with worse clinical outcomes^90^. In keeping with this, the significant associations between CD8^+^ TIL infiltration and worse prognosis in the studies described above were lost after adjusting for tumor grade. Similarly, the significant reduction in OS observed in the ER+/HER2− cancers in the BIG 02-98 analysis was not upheld in a multivariate analysis adjusting for other prognostic features^78^.

ILC is the second most common histological subtype of BC and is frequently ER+/HER2−. Compared to IDC, fewer data are currently available on the immune microenvironment in ILC^91^. Nevertheless, ILC has been shown to harbor lower TIL levels when compared to IDC^92,93^, although gene expression profiling has indicated that the transcriptomic immune response signatures may in fact be upregulated in ILC^94^. High TIL ILC has been associated with poor prognostic factors and, to date, emergent data suggest that high TILs are suggestive of less favorable clinical outcomes in ILC^95,96^. Indeed, it has been suggested that high TILs may drive aromatase inhibitor resistance which may in part account for some of the ILC-TIL signal identified^97,98^. However, the role of TILs in ILC is still unsolved, and more and larger, preferably pooled studies are needed to define the importance of TILs in this specific subtype.

The data on TIL levels and benefits from anti-estrogen endocrine therapy is even sparser. Sobral-Leite and colleagues evaluated CD8 expression in over 500 ER-positive BC patients who were randomized between no adjuvant endocrine treatments or one or three years of tamoxifen^88^. Patients with high CD8 infiltration in the tumor had a significant unfavorable prognosis; however, this effect was seen regardless of tamoxifen treatment. In addition, a study of 79 patients demonstrated a smaller reduction in tumor cell proliferation after two weeks of neoadjuvant aromatase inhibitor treatment in tumors with a significant lymphocytic infiltrate^91^. Another analysis from a prospective study of neoadjuvant letrozole ± lapatinib (*n* = 73) showed that significant Ki-67 reduction was observed in both patients with high and low baseline TILs, with high TILs tumors achieving more frequently a relative Ki-67 suppression >50% (55% vs. 35% of patients with low TILs)^99^. The long-term clinical significance of these observations remains unclear. There is also very limited data regarding the impact of targeted systemic therapy on TILs infiltration in ER-positive BC. Recently, CDK4/6 inhibitors have been reported to enhance antitumor immune responses associated with the upregulation of interferon-driven gene expression signatures^100^. However, this has not been associated with an increased TILs level in tumors^101^.

At present, there is no role for routine assessment of TILs in primary ER-positive BC, and their presence cannot be used to guide prognosis nor as a predictive biomarker. We propose that new studies should take a uniform approach to the assessment of TILs, such as those outlined in recent guidelines^23,102^ and should report data for luminal A and B tumors separately, In addition, a more refined understanding of the ER-positive BC immune environment, including the significance of immunosuppressive regulatory T cells, the spatial distribution of lymphocytes, and the role of estrogenic signaling will be required to define meaningful immune biomarkers in ER-positive disease^103–105^. Notably, a recent retrospective analysis (*n* = 563) of the multicenter IKA trial that randomized stage 1–3 ER-positive BC patients to receive tamoxifen or no adjuvant therapy showed that CD8-positive sTILs were significantly higher in patients with *PIK3CA*-mutated tumors (OR: 1.65; 95% CI: 1.03–2.68)^88^. In addition, this population of BC patients had an increased risk of recurrence (HR: 1.98; 95% CI: 1.14–3.41) on multivariate analysis as compared to those with CD4 and FOXP3 sTILs that were not statistically associated with prognostic outcomes^88^. This enrichment of TILs in tumors with mutated *PIK3CA*and their association with outcomes provided additional evidence on the crosstalk between mutational status and TME.

#### TILs in HER2-positive BC

Patients with HER2-positive early BC have a higher infiltration of TILs and improved pCR with NACT and trastuzumab^106,107^. Moreover, improved event-free survival in primary disease treated with trastuzumab and lapatinib was also noticed^107^. The predictive and prognostic value of TILs as a continuous variable in HER2-positive BC in the neoadjuvant setting was demonstrated in another analysis (*n* = 498) that evaluated TILs from two trials (GeparQuattro and GeparQuinto)^108^. In the group of patients with lymphocyte-predominant (≥60% sTILs) phenotype, a marked increase of pCR rates was noted^108^. Moreover, an increment of TILs by 10% was found to be an independent predictor of pCR in multivariate analysis (*p* = 0.014)^108^. Notably, this effect was more relevant in patients with ER+, PR+, and HER2+ tumors^108^. In the N9831 phase III trial (NCT00005970), tumor samples from patients with early HER2-positive BC were collected at baseline for TILs quantification in deciles with a prespecified categorical cutoff of ≥60%^109^. TILs were associated with recurrence-free survival in the cohort treated in the control arm with chemotherapy alone. Unexpectedly, higher TILs predicted a lack of response to trastuzumab in HER2 positive BC patients^109^. However, an immune gene-expression analysis on the same dataset did show a strong association with benefit^110^. A recent meta-analysis of 5 randomized and controlled trials that pooled data from 1256 patients provided additional evidence of the prognostic impact of TILs in the neoadjuvant setting^111^. Higher TILs at baseline were predictive of pCR irrespective of the neoadjuvant treatments used in HER2 positive BC^111^.

Recently, supporting evidence of the association of sTILs with distant DFS in this setting has emerged from the Short-HER phase III randomized study that compared different adjuvant regimens combined with trastuzumab (9 weeks vs. 12 months) (NCT00629278). On multivariate analysis, TILs predicted improved distant DFS for each 10% increment (HR: 0.73, 95% CI: 0.59–0.89, *p* = 0.006)^112^. At five years of follow-up, distant DFS rates were significantly higher in patients with TILs ≥ 20% (*p* = 0.025)^112^. Importantly, the 10% TIL increments were significantly associated with distant DFS only in patients randomized to receive nine weeks trastuzumab-based regimen (HR: 0.60, 95% CI: 0.41–0.88; *p* = 0.009)^112^.

In the National Surgical Adjuvant Breast and Bowel Project B-31 stromal TILs were also assessed^47^. Not only was the concordance between 6 different pathologists 90.8% when evaluated on a set of 100 representative cases from this trial, but sTILs were associated also with improved DFS. Higher sTILs were found associated with a higher response to trastuzumab-based on the 8-gene prediction model (*p* < 0.001)^47^. The association between TILs and prognosis in the advanced HER2-positive setting is less well established. A recent analysis of the pre-treatment samples from the CLEOPATRA study population revealed that higher quantities of TILs predicted an improved survival benefit in first-line treatment with taxane-based chemotherapy, trastuzumab, and pertuzumab^113^. Conversely, analysis of the samples from MA.31, a randomized clinical trial of lapatinib or trastuzumab in combination with taxane-based chemotherapy, reported that 35% of the population were categorized as high TIL but that this did not show significant prognostic or predictive effects^114^. It is worthwhile noting that the cutoff used for high TILs was 5%, much lower than the 20% cutoff used in the binary component of the CLEOPATRA analysis. MA.31 assessed TILs also on the primary tumor samples and found a mean of 9.2%; median, 5%; IQR, 1–10%. Provision of tumor tissue from a metastatic site pre and post-study treatment was an optional subsidy with low uptake—hence metastatic samples were not included in the TIL analysis. Conversely, the CLEOPATRA TILs analysis used samples of either the primary (93%) or metastatic (7%) sites and found the mean stromal TILs value was 21.07% and the median stromal TILs value was 10% (IQR 5–30). Although only a small number of metastatic samples were analyzed, the TILs values of samples from lung and lymph nodes were significantly higher than those from primary tumor, liver, bone, or skin. Furthermore, in the PANACEA-trial^115^, combined TILs and PD-L1 predicted benefit for pembrolizumab combined with trastuzumab in trastuzumab-resistant advanced HER2 positive BC. Whilst the early TNBC studies used a similar cutoff for high TIL groups, more recent analyses using TIL as a continuous variable has shown a linear association with survival outcomes. The ideal threshold for high TILs remains unclear in advanced HER2-positive disease, but variation in cut-offs may account for the conflicting results seen thus far^114^.

#### The incorporation of TILs into routine clinical practice and clinical trial design

It should be emphasized that formally speaking, the clinical utility of sTILs is not proven since no prospectively designed phase 3 TILs-driven trials have yet been performed, and evidence is lacking that treatment decisions based on sTILs favorably affect patient outcomes. Therefore, the *TILs Working Group* does not currently recommend the use of sTILs in isolation to guide treatment decisions in clinical practice. However, sTILs could be used to help identify patients with an excellent outcome to study *de-escalation* approaches and thereby reduce toxicity. In addition, based on the data of Luen et al., *escalation* of treatment regimens may be clinically beneficial for TNBC patients with a high post-neoadjuvant residual disease burden and low sTILs due to the poor prognosis^116^. Along those lines, the retrospective evaluation of sTILs in trials that have evaluated adjuvant treatments for those patients who did not reach a pCR, such as the CREATE-X trial—is planned^117^. In practice, some clinicians are using TILs *in conjunction* with other clinical variables to decide on the type and duration of cytotoxic chemotherapy. It may be emphasized that for most of the prognostic factors currently used for decades in BC daily practices, there is no level of 1A-evidence. To achieve clinical utility in TNBC, TILs must be included as pre-specified biomarkers in clinical trials and/or prospectively/retrospectively evaluated in randomized trials.

To successfully integrate TILs as a stratification factor in clinical trials, it is necessary to develop digital pathology tools to ensure accurate and rapid sTILs scoring by independent pathologists. Web-based platforms that allow easy scanning, viewing, and scoring of H&E slides should be optimized. A risk-based framework, aiming to reduce variability in TIL assessment within a clinical trial was used in the first stage of an adaptive non-comparative phase II trial (TONIC-trial)^118^. A total of 67 patients with metastatic TNBC were randomized to nivolumab (anti-PD-1) without induction or to one of four induction treatments, consisting of irradiation of one metastatic lesion (3 × 8 Gy) or a 2-week low-dose regimen of cyclophosphamide, cisplatin, or doxorubicin, all followed by nivolumab (NCT02499367)^118^. Concordance values between four pathologists were >90% in this trial, showing the applicability of this concept in real-world trial-settings^119^. The added value of web based-tools is that the biomarker scores of local pathologists can be integrated into the flow of a clinical trial, thereby enhancing local pathologists’ compliance and experience within a trial, bridging trial- with daily pathology practices. This is in contrast to the current situation, where the tissue may be sent to a central laboratory with limited to no involvement of local pathologists. Is the current trial-paradigm of “single-reference laboratory-approach” therefore in need of revision?

## PD-L1 testing and TILs in worldwide settings for BC

Access to novel therapeutic agents, including ICIs in low- and middle-income countries needs global support and importantly, standardization of testing. The “interchangeability of PD-L1 antibodies”, with a recent ESMO presentation showing all PD-L1 antibodies predict some benefit in TNBC for immunotherapy^71^ is still debated, illustrating the complexity and confusion that exists on these assays in the scientific community. In addition, the TILs Working Group, in a partnership with the College of American Pathologists, the European Society of Pathology, the Latin American Society of Pathology, and the European Working Group of Breast Screening Pathology argued how the current assay-approval policies, exemplified by the current PD-L1-assay situation, are leading to (unintended) imprecision medicine, which is not in the best interest of our patients. Hence, this pathology partnership proposed concrete solutions to improve the current assay approval pathway^120^. A framework for better evaluation of risks associated with suboptimal patient selection and stratification for ICI-based treatments in future clinical trials and real-world settings based on TILs/PD-L1 is proposed by the *TILs Working Group*^72^. The use of TILs as a predictive biomarker of response to ICI’s may considerably support cancer care in countries that have difficulty with PD-L1 implementation, mainly related to costs. Many ongoing clinical trials will report data that are expected to support TILs as a valid biomarker for response (see Table 2).

**Table 2:** Ongoing phase II/III clinical trials investigating TILs as a biomarker in invasive breast cancer.

Trial identifier and phase	Sample size	Setting	Purpose	Estimated primary completion date	Sponsor
NCT04188119 (IMpALA) Phase II	42	Triple-negative breast cancer (TNBC)	Assessment of post treatment tumor-infiltrating lymphocytes (TILs) by immunohistochemistry (as secondary outcome measure)	June 2021	The Christie NHS Foundation Trust
NCT03863301 (ABLATIVE-2) Phase II	70	Non-lobular invasive BC	Assessment TILs at baseline on biopsies and after surgery and their correlation with the pathological response (as a secondary outcome measure)	November 2022	UMC Utrecht, Netherland
NCT03820141 (DTP Trial) Phase II	39	HER2-amplified BC	Correlation of pathological complete response rate in BC patients with TILs (as secondary outcome measure)	March 2023	AstraZeneca
NCT03359954 Phase II	20	HR+/ HER2- BC	Evaluation of changes in TILs as a continuous variable before and after preoperative radiotherapy (RT) (as primary objective)	October 2019	M.D. Anderson Cancer Center
NCT03971045 (PERICLES) Phase II	46	Locally advanced “chest wall” BC	Positive PD-L1 (≥1%) and/or TILs (≥1%) as biomarkers for patient selection in locally advanced BC previously treated with chemotherapy or radiation therapy	July 2021	European Institute of Oncology
NCT02883062 Phase II	72	Newly diagnosed, stage II-III TNBC	Comparison of TILs percentage, PD-L1 and neoantigen load at baseline before and after therapy in patients receiving neoadjuvant chemotherapy (NACT) alone and those treated with NACT and atezolizumab in a randomized fashion	July 2020	National Cancer Institute (NCI)
NCT03366844 PhaseI/II	60	Invasive BC	TILs score changes (by Salgado *et al*. criteria)	January 2021	Cedars-Sinai Medical Center
NCT02435680 Phase II	50	Advanced TNBC with high tumor-associated macrophages (TAMs)	Assessment of TILs and TAMs content in pre- and post-dose tumor biopsies (as secondary outcome measure)	December 2019	Novartis Pharmaceuticals
NCT03894007 (PREDIX II HER2) Phase II	190	HER2-amplified early BC	TILs and gut microbiota as predictors of treatment response in patients receiving atezolizumab in a randomized fashion	December 2024	Karolinska University Hospital, Sweden
NCT03004183 (STOMP) Phase II	57	Metastatic TNBC	Measurement of the changes of TILs in tumor biopsy tissues as a biomarker of response to ADV/HSV-tk + valacyclovir therapy in combination with stereotactic body radiation therapy followed by immune-checkpoint blockade	May 2022	Merck Sharp & Dohme Corp.
NCT03979508 (BEAUTY Study) Phase II	100	Surgically resectable and chemotherapy-resistant TNBC	Quantification of TILs in TNBC patients treated with abemaciclib	July 2022	Mayo Clinic in collaboration with NCI
NCT02003209 Phase III	312	Locally advanced BC	Quantification of TILs in BC patients treated with neoadjuvant chemotherapy, trastuzumab, and pertuzumab with or without estrogen deprivation	August 2021	NCI
NCT02990845 (PEER) Phase I/II	25	Premenopausal HR+/ HER2- locally advanced or metastatic BC	Study of potentially predictive biomarkers of the efficacy of the combination of pembrolizumab and exemestane/leuprolide including TILs, PD-L1, circulating tumor cells, and mutational load	December 2019	National Taiwan University Hospital in collaboration with Merck Sharp & Dohme Corp.
NCT03125928 Phase II	50	Metastatic HER2-positive BC	Investigation of predictive biomarkers of response (PD-L1 and TILs) to immune-checkpoint blockade (as secondary outcome measure)	December 2020	Fox Chase Cancer Center in collaboration with Genentech, Inc.
NCT02971761 Phase II	29	Androgen receptor-positive metastatic TNBC	Evaluation of pre-treatment PD-L1 and TILs as predictive biomarkers of pembrolizumab and enobosarm	November 2020	City of Hope Medical Center in collaboration with NCI
NCT02849496	72	Locally advanced or metastatic non-HER2-positive BC	Measurement of TILs level changes in patients treated with olaparib with or without atezolizumab	August 2020	NCI
NCT02536794 Phase II	30	Metastatic HER2-negative BC	Evaluation of tissue-based immunohistochemical expression TILs, PD-L1, and other immune-related candidate biomarkers as predictors of response to MEDI4736 in combination with tremelimumab (as secondary outcome measure)	September 2019	Northwestern University in collaboration with MedImmune LLC Avon Breast Cancer Foundation and NCI
NCT03289819 Phase II	50	Early TNBC	Assessment of stromal and intratumoral TILs collected at baseline, 12 weeks after treatment initiation, and at the surgery	November 2019	Institute for Women’s Health in collaboration with Merck Sharp & Dohme Corp. And Celgene Corporation
NCT03740893 (PHOENIX) Phase II	81	NACT resistant and residual TNBC	Study of frequency and function of subsets of TILs in patients treated with DNA damage response inhibition and/or immune-checkpoint blockade (as secondary outcome measure)	May 2021	Institute of Cancer Research, the United Kingdom in collaboration with AstraZeneca

*ADV/HSV-tk* adenovirus-mediated herpes simplex virus thymidine kinase, *BC* breast cancer, *DNA* deoxyribonucleic acid, *HER2* human epidermal growth factor receptor, *HR* hormone receptor, *NACT* neoadjuvant chemotherapy, *PD-L1* programmed death-ligand 1, *RT* radiotherapy, *TAMs* tumor-associated macrophages, *TILs* tumor-infiltrating lymphocytes, *TNBC* triple-negative breast cancer. Data from ClinicalTrials.gov (accessed 15 December 2019).

### In Europe

In the United Kingdom (UK), the National Institute for Health and Care Excellence (NICE) reported that atezolizumab combined with nab-paclitaxel (Abraxane^®^) does not have plausible potential to be cost-effective in advanced TNBC. Nevertheless, PD-L1 testing and atezolizumab can be offered to patients with advanced TNBC. In Sweden, TILs have been incorporated in the Swedish Breast Cancer Guidelines as of June 2020^121^. Based on the results of Impassion 131, the New Therapies Council (NT-Rådet) does not recommend the use of atezolizumab in combination with paclitaxel or nab-paclitaxel for the treatment of advanced TNBC. The implementation of PD-L1 testing (SP142 assay) in Denmark has been hampered by the lack of analytical validity and reproducibility of immunostaining. Consequently, PD-L1 IHC is not in widespread use. In addition, the process concerning approval of atezolizumab for metastatic TNBC is ongoing in the Danish Medicines Council. TILs are included in the national Danish Breast Cancer Cooperative Group (DBCG) pathology guidelines. In Romania, PD-L1 testing is not routinely used. The testing of patients is made primarily by private pathology laboratories on request. National Health Insurance does not reimburse the bill for testing, so patients can enroll in a clinical trial or get free testing from the Pharmaceutical Industry accessing special testing programs. Atezolizumab is currently not approved for BC in Romania. Atezolizumab is approved and reimbursed in Italy for the treatment of patients with locally advanced or metastatic TNBC, and the SP142 assay has been identified as a companion diagnostic.

### In Africa

In Morocco, many breast pathologists practicing at university hospitals have benefited from the Roche^®^ training for the implementation of SP142 PD-L1 IHC. However, this assay has a high cost, and most of the national efforts to support cancer patients including health insurance do not cover this testing. Alternatively, several private pathology laboratories offer this assay on-demand with some oncological institutions providing access to ICI. Data from Kenya show HER-2 type BC prevalence of 17.6% and TNBC prevalence of 20.2%. Most patients with BC are still unable to meet the costs of expensive immunohistochemical PD-L1-testing and subsequent immunotherapy. In South Africa, PD-L1 testing is available on Dako and Ventana platforms but the cost is very high and not routinely performed in the public health sectors, which covers 75% of the population. In the private sector, PD-L1 testing is performed upon clinician request. The cost of ICIs drugs remains prohibitively high for use in the public sector. ICIs in BC are available only in the clinical trial setting, as these drugs are not licensed for use in TNBC. Assessment of TILs in BC care is currently not routinely done in either sector.

### In North and South America

In the United States, the PD-L1 SP142 assay has been approved as a companion assay for the selection of patients with TNBC to offer atezolizumab and taxane chemotherapy (http://www.svfp.se/foreningar/uploads/L15178/kvast/brostpatologi/Brostjuni2020.pdf). In Brazil, some central pathology laboratories perform PD-L1 testing based on principal investigator-sponsored agreements. PD-L1 IHC is tested mostly in private institutions with some programs offering pro bono testing to assess eligibility for approved ICI. The Brazilian Pathology Society follows WHO recommendations; hence the inclusion of TILs in daily practice can be envisaged in the near future. In Chile, PD-L1 testing is available on several platforms but their cost is high and this is not covered by public health insurance which covers 75% of the population. Anti-PD-L1 drugs remain at a high cost and are usually only funded by additional insurance or in clinical trial settings. In Argentina, PD-L1 testing is not covered by public funds. A few central private pathology laboratories perform PD-L1 testing on-demand funded by industry. Some pathologists have started to incorporate TILs into pathology reports for TNBC; however, there are no current national guidelines. In Perú, TILs evaluation has been included in TNBC protocols by pathologists in public and private centers. SP142 IHC is available in a few private labs while Dako 22C3 is being processed for non-breast malignancies in private and public centers. There are a few BC patients who have received atezolizumab funded via private insurance or clinical trials. It is not expected that the public system will support anti-PD-1 drugs in the near future.

#### In Asia

In India, PD-L1 and TIL assessments are not being performed routinely with significant discrepancies of results due to the different clones available to date. PD-L1 reporting is assessed on the platform as recommended by the standard reporting guidelines. TILs reporting is performed simultaneously with PD-L1 testing. Insurance companies do not offer reimbursement for PD-L1 testing. In April 2020, atezolizumab in combination with nab-paclitaxel received Drug Controller General of India (DCGI) approval as a first-line treatment for metastatic TNBC patients. This may pave the way for its use in selected patients if affordable (https://www.expresspharma.in/amp/latest-updates/roches-atezolizumab-receives-dcgi-approval-for-treatment-of-metastatic-triple-negative-breast-cancer-in-india/). OptiView PD-L1 (SP142) on the Ventana platform was approved in Japan as the companion diagnostic for metastatic TNBC treatment with atezolizumab. Other antibodies including 22C3, 28-8, and SP236 or OptiView PD-L1 (SP-142) on other platforms are un-funded. Pathology laboratories can use any PD-L1 assay for clinical research but only PD-L1-testing using SP142 on the Ventana platform companion diagnostic is permitted for funded treatment with atezolizumab in clinical practice. Many Japanese pathologists have limited clinical experience interpreting PD-L1 staining with no current national guidelines. The Japanese Society of Pathology does not provide oversight for quality control, which leaves this mostly to the pharmaceutical industry and individual laboratories. In Taiwan, PD-L1 assays are obligatory when considering immune checkpoint inhibition for BC. The fee for a PD-L1 assay is very high and the assay is not covered by the national health insurance and has to be paid at the patients’ own expense. The PD-L1 essay is only available in medical centers and some large hospitals.

In Malaysia, some ministry/government-based pathologists do not deem the PD-L1 interpretation as practical given the fact that immunotherapy is not generally provided to patients due to high costs and the lack of subsidized initiatives from the government. On the other hand, similar to that seen in Morocco, assays are being actively performed in the private setting with private health insurance covering the costs of the tests and the ICI. PD-L1 testing is not standardized in Iran, and this assay is not routinely requested in the setting of BC by oncologists. Additionally, sTILs are not routinely reported by pathologists in Iran, and those who do, use the method developed by the International TILS working group.

Cost issues are an extremely relevant topic for low-to-middle-income countries (LMIC) pertaining to PD-L1 testing. There is a considerable difference between the costing of the standard PD-L1-antibodies which are for most patients unaffordable. The high cost of PD-L1 assays in LMIC can be mitigated by selecting patients using the sTILs on H&E-slides to include cases for further analysis. Furthermore, as argued in detail before^72^, TILs and PD-L1 assays for immune cells are complementary as both are part of the same immune spectrum in cancer. However, the landscape of PD-L1 testing is clearly complex for both oncologists and pathologists. The guidelines for interpretation of immunohistochemical results as well as the selection of the appropriate companion diagnostic antibody vary with both tumor type and specific ICI under consideration. The Canadian evidence-based recommendations are a good example of the efforts provided to guide pathologists in establishing fit-for-purpose PD-L1 biomarker assays to select patients to benefit from ICIs in any tumor type^122^.

The International Immuno-Oncology Biomarker Working Group supports efforts to advance knowledge and best practices for immune-based therapies in all solid tumors. In this regard, the TIL Working Group has created an online tool (www.tilsinbreastcancer.org, “NEW PD-L1 Help Desk) to help oncologists and pathologists select and interpret appropriate anti-PD-1/PD-L1 therapies and associated companion diagnostics. This will be updated regularly to reflect the most current standards for eligibility for ICI for all solid tumors.

## A personal patient advocate perspective on TILs

“As a 5-year TNBC survivor who supports other BC patients to navigate and cope with their treatment, I know there’s no standard set of information that a patient uses to make their informed choice on how to treat their tumor. Some use Ki-67, some use PET scans; some are shown online risk tools like Predict, most use receptor status. Patients want as much information as possible to make the informed decisions forced upon them. We understand that nothing is “absolute”, that one hospital’s benchmark of a “clear margin” is different to another’s. We know that the thresholds defining hormone receptor subtypes differ between hospitals and that Ki-67 scoring can vary as much as three times between different pathologists. We understand the variability of response to drugs in different patients. We accept that pathology is an art but struggle to accept that cancer treatment plans arise from a random clutch of data-points and estimates of risk. Patients would benefit from owning their pathology reports. We live longer, we move between hospitals, and even when IT systems are compatible (which is rare) much of our data exist in unstructured reports that future clinicians do not access, either because they do not know it exists or they can’t find it amidst the hundreds of files that we generate. If patients had a checklist of markers that were important to their disease, we could help ensure evidence-based treatment plans. Patients suffer unnecessarily; it is stressful enough to face your own mortality but to trawl social media comparing your treatment to that of strangers creates more stress. Patient-ownership of pathology reports would help remove fears of age-bias and variation in care, and furthermore could help generate complete datasets for audits and real-world evidence studies.

We know that cancer is an ecosystem of cancer cells and the stromal cells between them. We know the well-defined markers for the cancer cells that direct chemotherapy and hormone therapy. What markers do we have for the intra-tumor stroma? PD-L1 SP142 is an expensive assay and notoriously difficult to achieve consensus on, but nevertheless if you ever once had PD-L1 recorded for any one of your TNBC primary tumors or metastases, you are eligible for one of the latest drugs, atezolizumab. Would it be wise therefore for all TNBC patients to request PD-L1 testing of their primaries for the event that their metastases can’t be biopsied? Shouldn’t we be seeking an easier and cheaper marker (e.g., TILs) to understand the stroma and direct treatment? I still feel very lucky that my TILs were scored in my pathology report. They have given me a more positive outlook and this alone is a reason for scoring them. I’m also very aware (through atezolizumab’s licensing) that details on my pathology report today might be my gateway to a life-prolonging drug tomorrow.”

## Conclusion

TILs are an inexpensive, robust prognostic biomarker that represents a surrogate for anti-tumor T cell-mediated immunity. Incorporating TILs into standard clinical practice should be strongly considered in both early and advanced TNBC and HER2-positive BC. TILs assessment at the time of diagnosis may enable a clinician to assess prognosis and in future inform therapeutic decision-making more accurately, is informative for predictive purposes, can help to interpret PD-L1 assays, and, certainly in LMIC, may be considered as a screening tool before embarking on expensive immune-assays, being PD-L1 or others. Further information on the clinical utility of TILs in HR positive BC is needed to identify their role in this subtype of the disease.

Education and standardization of testing across the pathology community by providing centralized training and educational tools are required to up-skill clinicians to utilize this new biomarker. The TIL-WG host’s pathologists from academic hospitals, community hospitals, and industry, supported by expert clinicians and statisticians, incorporating the view of patients also. We encourage transparent and efficient communication with the regulatory authorities. This collaboration of experts and patients is imperative to guide the development of this new biomarker and optimize its role across academia, industry, and the clinical setting. Looking to the future, a collaboration between the oncology and pathology communities across countries and continents is integral to define how best TILs can be integrated into a multivariate prognostic model with standard variables such as age, tumor size, and nodal status to optimize outcomes of patients with BC. In addition, trials being developed using baseline, on-treatment, and post-treatment TILs may improve our understanding of the complex interaction between host immunity and the TME and will improve our approach to fit as best as possible our patient’s needs.
